# Integration of FUNDC1-associated mitochondrial protein import and mitochondrial quality control contributes to TDP-43 degradation

**DOI:** 10.1038/s41419-023-06261-6

**Published:** 2023-11-11

**Authors:** Jinfa Ma, Lei Liu, Lu Song, Jianghong Liu, Lingyao Yang, Quan Chen, Jane Y. Wu, Li Zhu

**Affiliations:** 1 State Key Laboratory of Brain and Cognitive Science, Institute of Biophysics, Chinese Academy of Sciences, Beijing, 100101 China; 2 University of Chinese Academy of Sciences, Beijing, 100049 China; 3 State Key Laboratory of Membrane Biology, Institute of Zoology, Chinese Academy of Sciences, Beijing, 100101 China; 4 Interdisciplinary Center of Cell Response, State Key Laboratory of Medicinal Chemical Biology, College of Life Sciences, Nankai University, Tianjin, 300071 China; 5 Department of Neurology, Center for Genetic Medicine, Lurie Cancer Center, Northwestern University Feinberg School of Medicine, Chicago, IL 60611 USA

**Keywords:** Mechanisms of disease, Neurodegenerative diseases

## Abstract

Though TDP-43 protein can be translocated into mitochondria and causes mitochondrial damage in TDP-43 proteinopathy, little is known about how TDP-43 is imported into mitochondria. In addition, whether mitochondrial damage is caused by mitochondrial mislocalization of TDP-43 or a side effect of mitochondria-mediated TDP-43 degradation remains to be investigated. Here, our bioinformatical analyses reveal that mitophagy receptor gene *FUNDC1* is co-expressed with *TDP-43*, and both *TDP-43* and *FUNDC1* expression is correlated with genes associated with mitochondrial protein import pathway in brain samples of patients diagnosed with TDP-43 proteinopathy. FUNDC1 promotes mitochondrial translocation of TDP-43 possibly by promoting TDP-43-TOM70 and DNAJA2-TOM70 interactions, which is independent of the LC3 interacting region of FUNDC1 in cellular experiments. In the transgenic fly model of TDP-43 proteinopathy, overexpressing FUNDC1 enhances TDP-43 induced mitochondrial damage, whereas down-regulating FUNDC1 reverses TDP-43 induced mitochondrial damage. FUNDC1 regulates mitochondria-mediated TDP-43 degradation not only by regulating mitochondrial TDP-43 import, but also by increasing LONP1 level and by activating mitophagy, which plays important roles in cytosolic TDP-43 clearance. Together, this study not only uncovers the mechanism of mitochondrial TDP-43 import, but also unravels the active role played by mitochondria in regulating TDP-43 homeostasis.

## Introduction

TDP-43 proteinopathy is a group of neurodegenerative diseases characterized neuropathologically by the presence of protein inclusions containing phosphorylated TDP-43 protein [1]. This is a spectrum of genetically and clinically heterogeneous diseases, including amyotrophic lateral sclerosis (ALS-TDP), frontotemporal lobar degeneration (FTLD-TDP), Alzheimer’s disease [2] and recently defined Limbic Predominant Age Related TDP-43 Encephalopathy [3]. Mutations in the *TDP-43* gene have been identified only in a fraction of familial ALS-TDP and FLTD-TDP patients, but not in the majority of TDP-43 proteinopathy cases. Dysregulation of TDP-43 expression and protein homeostasis may contribute to the pathogenesis in most cases of TDP-43 proteinopathy.

Mitochondrial damage is an early event and an important aspect of the pathogenesis of TDP-43 proteinopathy [4–7]. A range of mitochondrial abnormalities have been reported in cellular and animal models of TDP-43 proteinopathy [4, 5, 8]. TDP-43 has been shown to impair mitochondrial Complex I [4, 5] and trigger mitochondrial DNA release to activate neuroinflammation [9]. It is known that TDP-43 enters mitochondria through TOM and TIM complex [4]. TOM-TIM is a common pathway to import most nuclear DNA-coded mitochondrial proteins into mitochondria. Some other proteins must play a regulatory role in the mitochondrial translocation of TDP-43 and protein homeostasis of TDP-43 inside neurons, which may also contribute to the neurotoxicity induced by TDP-43 dysregulation.

Increasing evidence supports the notion that mitochondria play important roles in protein homeostasis. Experimental evidence shows that some aggregation-prone proteins enter the mitochondria for degradation in yeast and mammalian cells [10–12]. Our previous study reveals that TDP-43 is translocated into mitochondria, activating mitochondrial unfolded protein response (UPR^mt^), and TDP-43 can be degraded by mitochondrial protease LONP1 [5]. Though it is widely accepted that TDP-43 can cause mitochondrial damage, whether the harmful effect is caused by the mislocalization of nuclear protein TDP-43 into mitochondria or by a side effect of mitochondria-mediated TDP-43 degradation remains to be investigated.

This study shows that mitochondria are vigorously involved in TDP-43 homeostasis, in which mitophagy receptor FUNDC1 plays important roles. Bioinformatical analyses reveal that *FUNDC1* gene is co-expressed with *TDP-43* and several members of HSP70/HSP40/TOM families in TDP-43 proteinopathy samples. Overexpressing TDP-43 increases FUNDC1 level, and FUNDC1 promotes the mitochondrial translocation of TDP-43, although these two proteins do not have a direct interaction. Biochemical experiments indicate that FUNDC1 and TDP-43 interact with TOM70, HSPA8, and DNAJA2, respectively, and the latter three proteins are associated with mitochondrial TDP-43 import. In the transgenic fly model of TDP-43 proteinopathy, altering FUNDC1 expression modifies TDP-43 induced mitochondrial damage. In addition, overexpressing FUNDC1 decreases cytosolic TDP-43 levels possibly by increasing LONP1 level and activating mitophagy. Together, our data support that FUNDC1 promotes the mitochondrial TDP-43 import by regulating proteins associated with the mitochondrial protein import pathway, and FUNDC1-mediated mitochondrial quality control plays an important role in TDP-43 homeostasis in TDP-43 proteinopathy.

## Results

### *FUNDC1* is co-expressed with HSP40, TOM, and TIM families at mRNA levels in TDP-43 proteinopathy

We analyzed publicly available GEO (https://www.ncbi.nlm.nih.gov/geo/) RNA-seq data from frontal cortex samples of 79 control subjects and of 195 TDP-43 proteinopathy patients (see “Methods” and Fig. S1 for detailed data collection and processing). We chose mitophagy receptors as focused targets because FUNDC1/HSPA8 (also known as HSC70) interaction boosts the mitochondrial translocation of unfolded cytosolic proteins for degradation [11]. Twelve mitophagy receptor genes were included, including *AMBRA1* [13], *ATAD3B* [14], *BCL2L13* [15], *BNIP3*, *BNIP3L* [16], *FKBP8* [17], *FUNDC1* [18], *OPTN* [19], *PHB2* [20], *NBR1* [21], *NDP52* [22], and *TAX1BP1* [23].

**Fig. 1:**
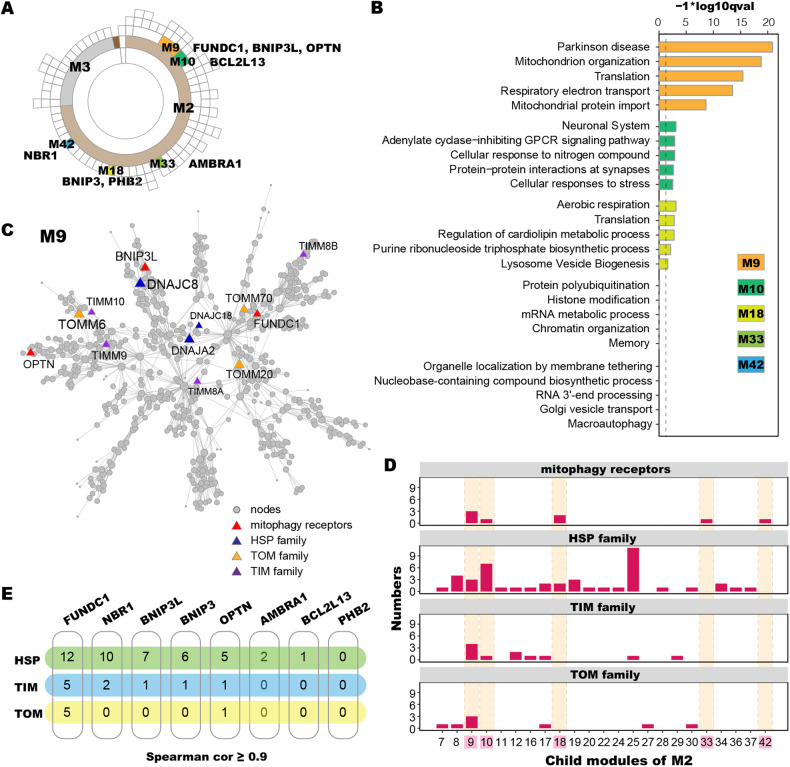
Co-expression network analyses of modules containing mitophagy receptor genes in RNA-seq data from TDP-43 proteinopathy samples. The co-expression network was constructed using MEGENA. **A** The hierarchical structure of co-expression network modules (M) in TDP-43 proteinopathy. Child modules of M2, including M9, M10, M18, M33, and M42, contain mitophagy receptor genes. **B** Gene ontology analysis by Metascape of nodes in M9, M10, M18, M33, and M42. **C** Co-expression network of M9. M9 contains a group of HSP, TOM, and TIM family members. **D** Gene numbers in different gene families (mitophagy receptor genes, HSP, TOM and TIM) in the child modules of M2. The modules containing mitophagy receptor, HSP, TOM, or TIM genes were visualized. **E** Expression correlation of different mitophagy receptor genes with HSP, TOM, and TIM family members. Those with Spearman correlation coefficient ≥ 0.9 are shown. The numbers represent gene numbers in corresponding gene families correlated with each mitophagy receptor gene.

Using the RNA-seq datasets, we constructed gene co-expression networks using MEGENA [24] in control and TDP-43 proteinopathy samples, respectively. Such analyses revealed remarkable reorganization of co-expression networks (Fig. 1A versus Fig. S2A). Co-expression network of patient samples unveiled five child modules (M) of M2 contained eight mitophagy receptor genes, i.e., M9 (*FUNDC1*, *BNIP3L*, and *OPTN*), M10 (*BCL2L13*), M18 (*BNIP3* and *PHB2*), M33 (*AMBRA1*) and M42 (*NBR1*) (Fig. 1A). Gene pathway enrichment analyses showed that M9 was associated with mitochondrial protein import (Fig. 1B). This module also contained HSP40 (DNAJ), TOM and TIM family members (Fig. 1C), which play important roles in the mitochondrial protein import. Among different child modules of M2, only M9 contained all four gene families, i.e., mitophagy receptor, HSP, TOM, and TIM family members, as its nodes (Fig. 1D). Spearman correlation coefficients showed that the expression of *FUNDC1* was positively correlated with more HSP, TOM, and TIM family members than other mitophagy receptor genes (Fig. 1E). In control samples, TOM or TIM family members were not found in the FUNDC1-containing module. Instead, a number of HSP family members, especially HSP40 family members, were identified in this module (Figs. S2A and S2B). These results suggest that FUNDC1 together with proteins associated with mitochondrial protein import (TOM, TIM, and HSP40 family members) may play potential roles in TDP-43 proteinopathy.

### FUNDC1 promotes mitochondrial translocation of TDP-43

**Fig. 2:**
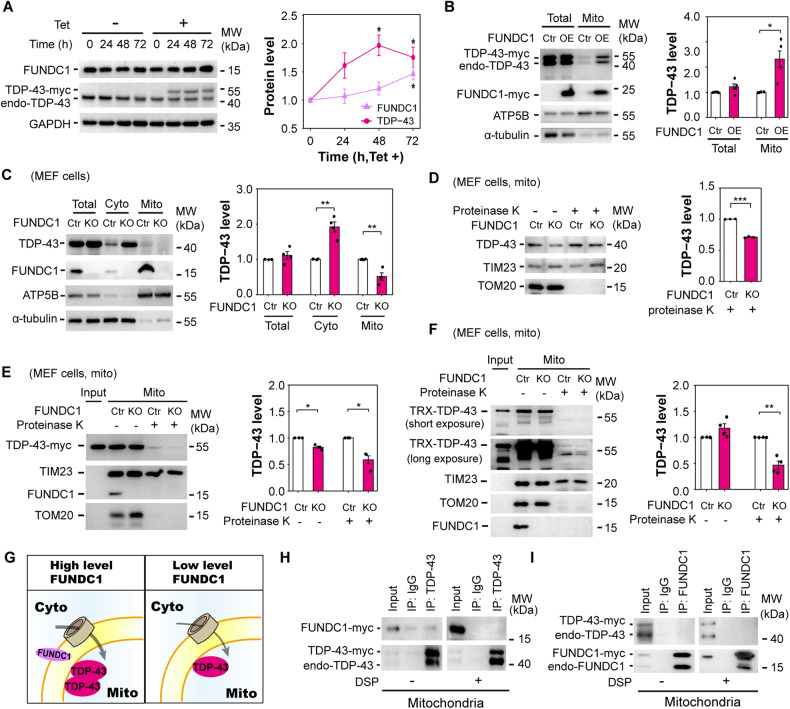
FUNDC1 promotes mitochondrial translocation of TDP-43. **A** Immunoblotting and quantification of FUNDC1 level after overexpressing TDP-43. Tetracycline-inducible HEK293 cells stably expressing myc-his-tagged TDP-43 were treated with tetracycline (Tet, 1 μg/mL) to induce TDP-43 overexpression. The combined exogenous and endogenous TDP-43 was used for quantification. GAPDH was loaded as a reference. **B** Immunoblotting and quantification of total and mitochondrial TDP-43 levels after overexpressing FUNDC1. Tetracycline-inducible HEK293 cells (in 10 cm dishes) were transfected with 4 μg pcDNA4 TO plasmids expressing FUNDC1 using VigoFect. After 12 h of transfection, tetracycline (1 μg/mL) was added to induce TDP-43 overexpression for another 48 h. The combined exogenous and endogenous TDP-43 was used for quantification. α-tubulin and ATP5B were loaded as total and mitochondrial references, respectively. **C** Immunoblotting and quantification of total, cytosolic and mitochondrial TDP-43 levels in FUNDC1 KO MEF cells. α-tubulin was loaded as total and cytosolic reference, and ATP5B was loaded as mitochondrial reference. **D** Immunoblotting and quantification of mitochondrial TDP-43 level after proteinase K digestion in FUNDC1 KO MEF cells. TIM23 was loaded as a reference. **E**, **F** Immunoblotting and quantification of myc-his-tagged TDP-43 (**E**) and TRX-his-tagged TDP-43 (**F**) levels after mitochondrial import assay. TIM23 was loaded as a mitochondrial reference. **G** A working model showing that FUNDC1 is required for efficient mitochondrial TDP-43 import. **H**, **I** Co-IP of TDP-43 and FUNDC1. The cells were treated with or without crosslinker, DSP, and then Co-IP experiments were performed using purified mitochondria with anti-TDP-43 antibody (**H**) or anti-FUNDC1 antibody (**I**) followed by immunoblotting using corresponding antibodies as indicated. Data in panels (**A**–**F**) are presented as mean ± SEM and analyzed by Student’s *t*-test (**A**, **D**, **E**: *n* = 3 independent experiments; **B**, **C**, **F**: *n* = 4; ***P < 0.001, **P < 0.01, *P < 0.05).

To test the role of FUNDC1 in regulating mitochondrial TDP-43 import, we examined mitochondrial TDP-43 levels in tetracycline-inducible HEK293 cells stably expressing myc-his tagged TDP-43 [5] or FUNDC1 knockout (KO) mouse embryonic fibroblast (MEF) cells [25]. FUNDC1 level was continuously increased after inducing exogenous TDP-43 expression for 72 h (Fig. 2A). Interestingly, overexpressing FUNDC1 increased mitochondrial protein levels of both exogenous and endogenous TDP-43 proteins (Fig. 2B). In FUNDC1 KO MEF cells, mitochondrial TDP-43 level was decreased and cytosolic TDP-43 level was increased (Fig. 2C). Furthermore, proteinase K protection experiments showed that the level of TDP-43 protein inside mitochondria was significantly decreased in FUNDC1 KO cells (Fig. 2D). In addition, in vitro mitochondrial import assay showed that either myc-his-tagged TDP-43 purified from Tet-on HEK293 cells or TRX-his-tagged TDP-43 purified from bacteria was significantly decreased in mitochondrial fraction prepared from FUNDC1 KO MEF cells (Fig. 2E, F). These data demonstrate that FUNDC1 promotes mitochondrial translocation of TDP-43 (Fig. 2G). However, no obvious interaction was detected between FUNDC1 and TDP-43 in the co-immunoprecipitation (Co-IP) experiments (Fig. 2H, I). Thus, FUNDC1 may promote mitochondrial translocation of TDP-43 in an indirect manner, probably with the assistance of other factors. We then detected the possible candidates with the guidance of gene co-expression analysis.

### Both *FUNDC1* and *TDP-43* are co-expressed with *HSPA8*/*A9*/*A5*

Although no obvious direct interaction was detected between FUNDC1 and TDP-43 proteins, *FUNDC1* and *TDP-43* expression were positively correlated at mRNA levels no matter in disease or control samples (Fig. S3A). Co-expression network analyses revealed that *FUNDC1* was co-expressed with HSP40s (Figs. 1C and S2), co-chaperones of HSP70s. A recent study reported that TDP-43 interacted with HSP70 family members, such as HSPA1A, HSPA5, HSPA6, and HSPA8 [26]. In addition, a subset of HSP70 family members has been reported to interact with FUNDC1, including HSPA1A, HSPA2, HSPA6, HSPA8, and HSPA9 (mitochondrial matrix HSP70) [11]. Spearman correlation coefficients showed that both *TDP-43* and *FUNDC1* were positively correlated with *HSPA5*, *HSPA8*, and *HSPA9* at the mRNA levels either in the GEO datasets (Fig. S3B) or in the GTEx (www.gtexportal.org) dataset (Fig. S3C), which was mainly observed in the central nervous system (Fig. S3C). Allen Brain (https://portal.brain-map.org) dataset showed that *TDP-43*, *HSPA5*, *HSPA8*, *HSPA9*, and *FUNDC1* were all highly expressed in neurons but not in other cell types of neural tissues (Fig. S3D). These data indicate that both *FUNDC1* and *TDP-43* are co-expressed with *HSPA8*/*A9*/*A5*. Among the three HSP70 genes, altering HSPA8 or HSPA9 expression affected mitochondrial TDP-43 levels in the transgenic Drosophila model expressing human TDP-43 [5, 27] (Fig. S4). Therefore, HSPA8 and HSPA9 were selected as the candidates in the following study.

### Both TDP-43 and FUNDC1 interact with HSPA8, DNAJA2, and TOM70

*FUNDC1* was co-expressed with *DNAJA2*, *DNAJC8*, and *DNAJC18* (Figs. 1C, S5A, S5C, and S5E). These three HSP40 genes were also co-expressed with *TDP-43* (Figs. S5A, S5C, and S5E) and were all highly expressed in neurons (Figs. S5B, S5D, and S5F), similar to HSP70 genes (Fig. S3B-D). *DNAJA2* (R_dis_ = 0.96) was at the top of the three HSP40 genes that were positively correlated with *FUNDC1* expression in TDP-43 proteinopathy (Figs. S5A, S5C, and S5E). DNAJA2 is associated with mitochondrial protein import [28]. Therefore, DNAJA2 was selected as a candidate. In addition, TOM20 and TOM70 (whose encoding genes were in the FUNDC1 containing M9, Fig. 1C), as well as TOM channel-forming subunit TOM40, were also included in the study.

**Fig. 3:**
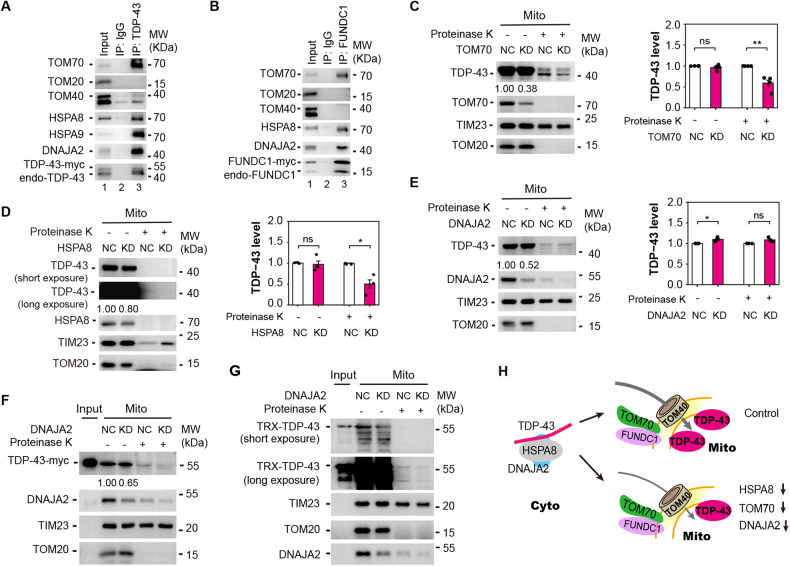
FUNDC1 and TDP-43 interact with HSPA8, DNAJA2, or TOM70, and down-regulating HSPA8, DNAJA2, or TOM70 decreases mitochondrial TDP-43 level. **A** Co-IP of TDP-43 and TOM70, HSPA8, DNAJA2, or HSPA9 (mt-HSP70). **B** Co-IP of FUNDC1 and TOM70, HSPA8 and DNAJA2. Mitochondria were prepared from tetracycline-inducible HEK293 cells (**A**) and HEK293 cells overexpressing FUNDC1 (**B**) for co-IP (**A**, **B**). **C**–**E** Immunoblotting and quantification of mitochondrial TDP-43 level after knocking down of TOM70 (**C**), HSPA8 (**D**), or DNAJA2 (**E**). TDP-43 level relative to TIM23 is quantified, and data are presented as mean ± SEM and analyzed by Student’s *t*-test (**C**: *n* = 4 independent experiments; **D**, **E**: *n* = 3; **P < 0.01, *P < 0.05, ns: no significance). **F**, **G** Immunoblotting of myc-his-tagged TDP-43 (**F**, representative results of three independent experiments) or TRX-his-tagged TDP-43 (**G**, representative results of three independent experiments) in vitro mitochondrial import assay using mitochondria purified from cells with DNAJA2 knockdown. **H** A diagram showing that down-regulating HSPA8, TOM70 or DNAJA2 decreases inner mitochondrial TDP-43 level.

Co-IP experiment showed that TDP-43 interacted with TOM70, TOM40, HSPA8, HSPA9, and DNAJA2, respectively (Fig. 3A), and FUNDC1 interacted with TOM70, HSPA8 and DNAJA2, respectively (Fig. 3B). Down-regulating either TOM70 or HSPA8 decreased mitochondrial TDP-43 level (Fig. 3C, D). It should be noted that the results of HSPA8 knockdown in cells (Fig. 3D) were different from those in HSPA8 knockdown files (Fig. S4). One possible explanation is that flies were exposed to not only TDP-43 overexpression but also Hsc70-4 knockdown under the periodic heat shock (see Methods for details), whereas the cell model was only exposed to continuous HSPA8 knockdown. When flies are under heat shock, the mitochondrial TDP-43 import might be impaired, which causes cytosolic accumulation of overexpressed TDP-43. After heat shock, accumulated cytosolic TDP-43 might be translocated into mitochondria thereby increasing mitochondrial TDP-43 level. Though down-regulating DNAJA2 did not affect mitochondrial TDP-43 level possibly caused by the compensation effect of its paralogs DNAJA1 and DNAJA4 in cells [29] (Fig. 3E), the ability to translocate TDP-43 by mitochondria purified from DNAJA2 knockdown cells was decreased, shown by in vitro mitochondrial import assay (Fig. 3F, G). Therefore, down-regulating either one of three genes (*TOM70*, *HSPA8*, *DNAJA2*) affects the mitochondrial TDP-43 import (Fig. 3H).

### FUNDC1 enhances DNAJA2-TOM70 and TDP-43-TOM70 interactions

**Fig. 4:**
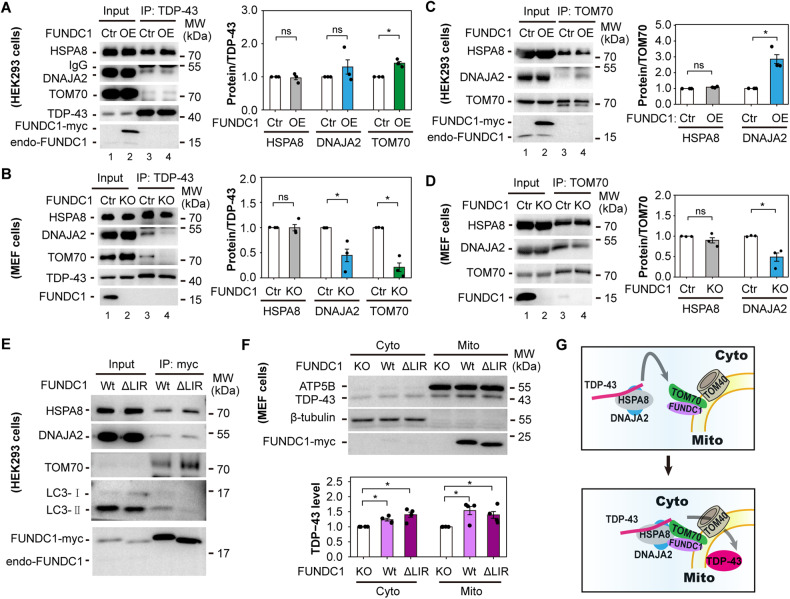
FUNDC1 enhances TDP-43-TOM70 and DNAJA2-TOM70 interactions independent of the LIR motif. **A**, **B** Co-IP of TDP-43 with HSPA8, DNAJA2 or TOM70 in HEK293 cells overexpressing (OE) FUNDC1 (**A**) and in FUNDC1 KO MEF cells (**B**). Protein level relative to TDP-43 was quantified. **C**, **D** Co-IP of TOM70 with HSPA8 or DNAJA2 in FUNDC1 OE HEK293 cells (**C**) and in FUNDC1 KO MEF cells (**D**). Protein level relative to TOM70 was quantified. HEK293 cells (in 10 cm dishes) were transfected with 4 μg pcDNA4 TO plasmids expressing FUNDC1 using VigoFect. After 48 h of transfection, cells were harvested for co-IP in (**A**) and (**C**). Total cell lysate was prepared for co-IP in (**A**–**D**). **E** Co-IP of wild type (Wt) FUNDC1 and FUNDC1 LC3 interacting region deletion mutant (ΔLIR) with HSPA8, DNAJA2, TOM70 and LC3. HEK293 cells (in 10 cm dishes) were transfected with 4 μg pcDNA4 TO plasmids expressing FUNDC1-Wt or FUNDC1-ΔLIR using VigoFect. After 48 h of transfection, cells were harvested and total cell lysate was prepared for co-IP. **F** Immunoblotting and quantification of cytosolic and mitochondrial TDP-43 levels after overexpressing FUNDC1-Wt and FUNDC1-ΔLIR in FUNDC1 KO MEF cells. FUNDC1 KO MEF cells (in 10 cm dishes) were transfected with 8 μg pcDNA4 TO plasmids expressing FUNDC1-Wt or FUNDC1-ΔLIR using lipofectamine^TM^ 3000. After 18 h of transfection, cells were harvested for protein analysis. Data in (**A**–**D**) and (**F**) are presented as mean ± SEM and analyzed by Student’s *t*-test (**A**–**D**: *n* = 3 independent experiments; **F**: *n* = 4; *P < 0.05, ns: no significance). **G** A working model showing the mechanism of how FUNDC1 promotes mitochondrial translocation of TDP-43.

We examined the interaction of TDP-43 with TOM70, DNAJA2, or HSPA8 [11] after altering FUNDC1 expression. Overexpressing (OE) FUNDC1 increased TDP-43-TOM70 interaction (Fig. 4A); whereas knocking out (KO) FUNDC1 decreased both TDP-43-TOM70 and TDP-43-DNAJA2 interactions (Fig. 4B). Furthermore, TOM70-DNAJA2 interaction was increased by overexpressing FUNDC1 and decreased by knocking out FUNDC1 (Fig. 4C, D). Intriguingly, deleting the LC3 interacting region (LIR) of FUNDC1 did affect the FUNDC1-LC3 interaction but did not affect the interaction between FUNDC1 and HSPA8, DNAJA2, or TOM70 (Fig. 4E). Furthermore, overexpressing either wild type FUNDC1 or FUNDC1 LIR deletion mutant increased mitochondrial TDP-43 levels in FUNDC1 KO MEF cells (Fig. 4F).

Taken together, it is likely that FUNDC1 promotes mitochondrial TDP-43 import by enhancing TDP-43-TOM70 and TOM70-DNAJA2 interactions, which is independent of its LIR motif. Such enhanced interactions might promote the recognition of the TDP-43/HSPA8/DNAJA2 complex by TOM70 and further promote TDP-43 entering TOM channel (Fig. 4G).

### Overexpressing FUNDC1 enhances TDP-43-induced mitochondrial damage whereas knockdown of FUNDC1 reverses the mitochondrial damage in TDP-43 flies

**Fig. 5:**
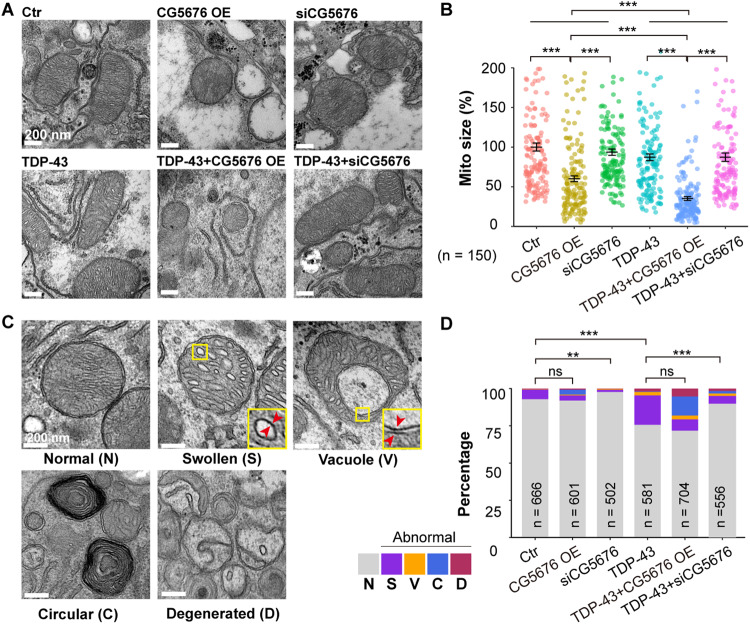
Overexpressing fly FUNDC1 (CG5676) enhances TDP-43 induced mitochondrial impairment, whereas down-regulating FUNDC1 partially reverses mitochondrial damage. **A**, **B** Representative TEM images (**A**) and quantification of mitochondrial size (**B**) in different groups. Ctr (control), CG5676 OE (CG5676 overexpression), siCG5676 (CG5676 knockdown), TDP-43 (TDP-43 overexpression), TDP-43+CG5676 OE, and TDP-43+siCG5676. Data are presented as mean ± SEM and analyzed by Two-way ANOVA followed by a post hoc test (*n* = 150 images from 3 individual flies in each group; ***P < 0.001, ns: no significance). Scale bars**:** 200 nm. **C** Mitochondrial morphological changes as detected by TEM. Normal (N) and four types of abnormal mitochondria were detected: mitochondria with swollen cristae (S), mitochondria with circular cristae (C), mitochondria with vacuole (V), and degenerated mitochondria (D). Compared with type S mitochondria, type V mitochondria contain vacuole(s) with a double-layered membrane, as shown in zoomed-in pictures (red arrowheads). Scale bars: 200 nm. **D** Quantification of normal and total abnormal mitochondria in different groups. Male flies were used in the experiments. Data are analyzed by the Chi-square test with Bonferroni correction for multiple comparisons (***P < 0.001, **P < 0.01, ns: no significance). Fly genotypes: Ctr: GMR-Gal4/tub-Gal80^ts^/UAS-RFP; CG5676 OE: GMR-Gal4/tub-Gal80^ts^/UAS-orfCG5676-HA; siCG5676: GMR-Gal4/tub-Gal80^ts^/UAS-siCG5676; TDP-43: GMR-Gal4/tub-Gal80^ts^/UAS-TDP-43-RFP-HA/attp40; TDP-43+siCG5676: GMR-Gal4/tub-Gal80^ts^/UAS-TDP-43-RFP-HA/UAS-siCG5676; TDP-43+CG5676 OE: GMR-Gal4/tub-Gal80^ts^/UAS-TDP-43-RFP-HA/UAS-orfCG5676-HA.

We then examined the effect of FUNDC1 on the morphological structures of mitochondria in male TDP-43 flies using transmission electron microscopy (TEM) since male ALS mice and male TDP-43 flies showed more aggressive manifestations than females [5, 30]. Under our experimental condition (Fig. S6A), TEM images showed that overexpressing fly FUNDC1 (CG5676) decreased the mitochondrial size whereas overexpressing TDP-43 had little effect on the mitochondrial size compared to control flies (Fig. 5A, B). However, overexpressing FUNDC1 further decreased the mitochondrial size in TDP-43 flies, whereas down-regulating fly FUNDC1 showed no further effect under TDP-43 background (Fig. 5B). After analyzing the control groups (Ctr, CG5676 OE, siCG5676) and TDP-43 overexpressing groups (TDP-43, TDP-43 + CG5676 OE, TDP-43+siCG5676), the comparison test revealed a significant difference (analyzed by Two-way ANOVA followed by a host hoc test, Fig. 5B).

TEM images revealed five mitochondrial types: normal mitochondria (N), and four types of abnormal mitochondria, including those with swollen cristae (S) or circular cristae (C) or containing vacuole (V) or losing cristae (also known as degenerated (D) mitochondria, Fig. 5C). Overexpressing TDP-43 significantly increased the percentage of abnormal mitochondria (Fig. 5D), especially type S mitochondria, consistent with our previous study [5]. Overexpressing fly FUNDC1 did not affect the percentage of total abnormal mitochondria in TDP-43 flies (Fig. 5D). However, overexpressing fly FUNDC1 increased the percentage of type C mitochondria with enriched protein aggregates [11] (Chi-square test, *P* < 0.001) and type D mitochondria (Chi-square test, *P* < 0.05) with severe damage in TDP-43 flies (Fig. 5D). Interestingly, down-regulating FUNDC1 restored the percentage of normal mitochondria (Fig. 5D) although it had no effect on the mitochondrial size (Fig. 5B). Therefore, up-regulating fly FUNDC1 enhances TDP-43-induced mitochondrial damage, whereas down-regulating fly FUNDC1 rescues TDP-43-induced mitochondrial damage. As expected, the effect of fly FUNDC1 knockdown was consistent with the function of FUNDC1 in promoting mitochondrial translocation of TDP-43 (Fig. S6B, C). Unexpectedly, FUNDC1 promoted mitochondrial TDP-43 import in a time-dependent manner (Fig. S6B, C versus Fig. S6E, F), possibly at an early time point after induction of TDP-43 expression (Fig. S6A versus Fig. S6D).

### FUNDC1 regulates mitochondria-mediated TDP-43 degradation

**Fig. 6:**
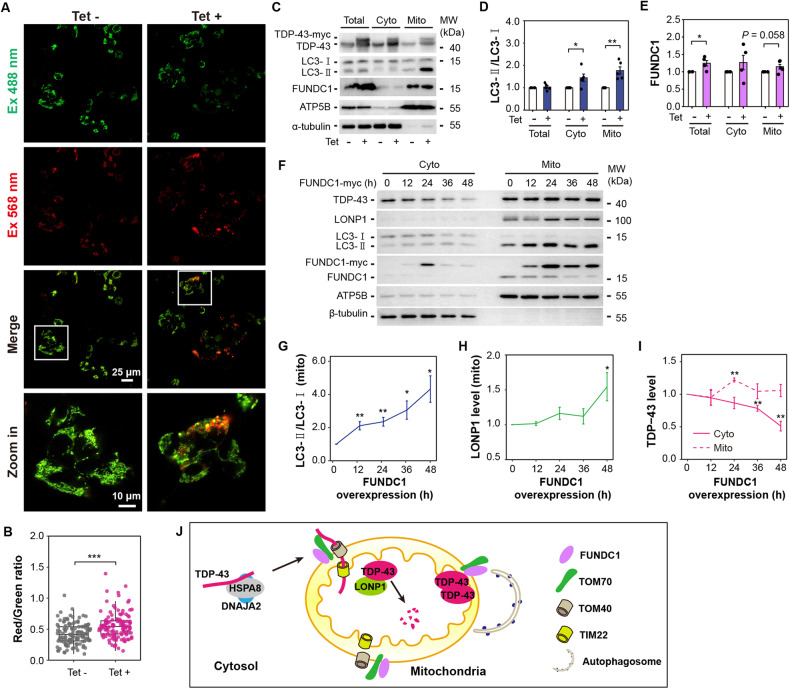
Overexpressing TDP-43 activates mitophagy, and FUNDC1-associated mitochondrial quality control contributes to the cytosolic TDP-43 clearance. **A** Representative fluorescence images of mito-Keima in tetracycline-inducible HEK293 cells after inducing TDP-43 overexpression with tetracycline (1 μg/mL) for 72 h. Scale bar: 25 μm, or 10 μm in zoomed-in image. **B** Quantification of the ratio between red and green signals in (**A**). Data are analyzed by One-way ANOVA followed by a post hoc test (*n* = total 120 images from 3 independent experiments in each group; ***P < 0.001). **C**–**E** Immunoblotting (**C**) and quantification of LC3 (**D**) and FUNDC1 (**E**) levels after inducing TDP-43 overexpression for 72 h in tetracycline-inducible HEK293 cells. α-tubulin and ATP5B were loaded as cytosolic and mitochondrial references. **F**. Immunoblotting of TDP-43, LONP1, and LC3 after overexpressing FUNDC1 for 12, 24, 36, and 48 h. Neuronal SH-SY5Y cells (in 10 cm dishes) were transfected with 8 μg pcDNA4 TO plasmids expressing wild-type FUNDC1 using lipofectamine^TM^ 3000. β-tubulin and ATP5B were loaded as cytosolic and mitochondrial references. **G**–**I** Quantification of mitochondrial LC3 level (**G**), mitochondrial LONP1 level (**H**), cytosolic and mitochondrial TDP-43 levels (**I**) from immunoblotting in (**F**). Data in (**G**–**I**) are presented as mean ± SEM and analyzed by a Student’s *t*-test (*n* = 5 independent experiments; **P < 0.01, *P < 0.05). **J** A working model showing that FUNDC1 promotes mitochondrial TDP-43 import and regulates mitochondria-mediated TDP-43 degradation.

The time-dependent manner of mitochondrial TDP-43 level changes after overexpressing fly FUNDC1 indicates that mitochondria-mediated protein degradation processes might be activated. Overexpressing TDP-43 in flies resulted in the activation of mitophagy observed by TEM images (Fig. S7). Activated mitophagy was also detected after induction of TDP-43 expression in HEK293 Tet-on cells by mito-Keima fluorescence change and increased LC3-II/LC3-I ratio (Fig. 6A–D). Consistently, overexpressing TDP-43 increased mitochondrial FUNDC1 level (Fig. 6E). Inhibition of either ubiquitin-proteasome system (UPS) or mitochondrial protease LONP1, two processes that are associated with TDP-43 degradation [5, 31], increased mitochondrial TDP-43 level and further activated mitophagy (Fig. S8A–C). Furthermore, mitochondrial FUNDC1 level was decreased and cytosolic FUNDC1 was increased after inhibiting UPS (Fig. S8D). Changes in subcellular FUNDC1 level might result from the mitophagy process in which damaged mitochondria segregate from the mitochondrial network and undergo degradation by lysosomal proteases [32]. Therefore, FUNDC1 may have a dual role in TDP-43 degradation, acting not only through its involvement in facilitating mitochondrial import of TDP-43 but also potentially mediating mitophagy to promote TDP-43 clearance.

TDP-43 proteinopathy represents a group of neurodegenerative disorders characterized by progressive neuronal damage, culminating in the eventual death of damaged neurons. To investigate the role of FUNDC1 in TDP-43 degradation in neuron-like cells, we performed a longitudinal assessment of FUNDC1 overexpression in human SH-SY5Y neuroblastoma cells (Fig. 6F). With the FUNDC1 overexpression, the mitochondrial LC3-II/LC3-I ratio was gradually increased (Fig. 6F, G). LONP1 level was nearly stable after overexpressing FUNDC1 for 36 h but significantly increased when expressing FUNDC1 for 48 h (Fig. 6F, H). Meanwhile, cytosolic TDP-43 was gradually decreased (Fig. 6F, I). Such results indicate that overexpressing FUNDC1 contributes to cytosolic TDP-43 clearance. Consistent with the results in TDP-43 flies, mitochondrial TDP-43 did not constantly increase but started to decrease after overexpressing FUNDC1 over 24 h (Fig. 6I). Such time-dependent changes of TDP-43 levels were also observed in FUNDC1 KO MEF cells when overexpressing wild type FUNDC1 or LIR deletion mutant (Fig. S9). Decreased mitochondrial TDP-43 might be caused by decreased cytosolic TDP-43 level and increased mitochondria-mediated TDP-43 degradation by either LONP1 or mitophagy (Fig. 6).

## Discussion

TDP-43 enters mitochondria through the common mitochondrial import pathway composed of TOM and TIM complex [4]. However, some other factors must play regulatory roles in this process. In the present study, we show that *FUNDC1* is co-expressed with genes critical for mitochondrial protein import, such as *HSPA8*, *TOM70*, and *DNAJA2* (Figs. 1, S3, and S5), and FUNDC1 regulates mitochondrial TDP-43 import (Figs. 2, S6, and S9) and mitochondria-mediated TDP-43 degradation (Fig. 6). Based on the results of this study, a working model of FUNDC1’s dual role in regulating mitochondrial translocation of TDP-43 and mitochondria-mediated TDP-43 degradation is proposed and shown in Fig. 6J.

FUNDC1 is required for efficient mitochondrial translocation of TDP-43 (Fig. 2). FUNDC1 interacts not only with HSPA8/DNAJA2 but also with TOM70 (Fig. 3B), a component of the TOM complex that is important for the mitochondrial import of proteins lacking classical mitochondrial targeting sequences [33]. TOM70 contains tetratricopeptide repeats (TPRs), and the TPRs mediate the binding between TOM70 and cytosolic chaperones, including HSPA8 [34]. DNAJA2 is a co-chaperone of HSPA8 and is closely related to DNAJA1 and DNAJA4 [35], and these DNAJAs are important for TOM70-mediated mitochondrial protein import [29]. It has been proposed that Ydj1, a yeast homolog of DNAJAs [35, 36], may help to target preprotein-HSP70 complexes to the mitochondrial outer membrane [36]. Our data showed that FUNDC1 increased DNAJA2-TOM70 interaction (Fig. 4C, D) and TDP-43-TOM70 interaction (Fig. 4A, B). Thus, FUNDC1 may facilitate targeting the HSPA8/DNAJA2/TDP-43 complexes to TOM70 (Fig. 4G), thereby promoting mitochondrial TDP-43 import.

A previous study showed that the mature parts of the TOM70-dependent mitochondrial precursor proteins have the propensity to aggregate [37], similar to TDP-43 [31]. Integrating mitochondrial protein import and mitochondria-mediated protein degradation may closely regulate these aggregation-prone proteins, in which FUNDC1 might play important roles. On one hand, FUNDC1 promotes mitochondrial TDP-43 import independent of its LIR motif (Fig. 4E, F). Increased mitochondrial TDP-43 import enhances LONP1 level (Fig. 6H), thereby promoting LONP1-mediated protein degradation. This is consistent with our previous study that TDP-43 expression activates UPR^mt^ and mitochondrial TDP-43 can be degraded by LONP1 [5], supporting that LONP1 protects against TDP-43-induced mitochondrial damage, and LONP1 is indeed up-regulated in FTLD-TDP patients [5]. On the other hand, FUNDC1 is a mitophagy receptor, which can trigger mitophagy and contribute to TDP-43 degradation (Figs. 6, S7, and S8). However, when the mitochondrial TDP-43 level exceeds a certain threshold for clearance, it may result in mitochondrial damage, thereby causing excessive mitophagy, and excessive mitophagy will lead to neurotoxicity [38, 39] (see the effect of continuous FUNDC1 and TDP-43 overexpression in files in Fig. 5). FUNDC1 is down-regulated in patients with TDP-43 proteinopathy (Fig. S3A, detected by DESeq2, adjust *P* value < 1 × 10^−10^), suggesting a potential protective mechanism against excessive mitochondrial protein import [40] during the advanced stages of the disease. However, this protective role (Fig. 5D) may inadvertently contribute to the accumulation of cytosolic TDP-43 (Fig. 2C), which is one of the most predominant pathological features observed in TDP-43 proteinopathy.

In conclusion, our study not only uncovers the critical role of the mitophagy receptor in regulating mitochondrial TDP-43 import but also advances the understanding of the mechanism for maintaining TDP-43 homeostasis in neuronal cells. Balancing mitochondrial proteostasis and mitochondria-mediated protein degradation induced mitochondrial damage might provide therapeutic strategies for the treatment of TDP-43 proteinopathy.

## Materials and methods

### Fly strains

Flies used for hybridization are listed in Table S1. All flies were raised in standard fly food, 50% relative humidity, and 12–12 h light-dark cycles. Unless specified, otherwise, flies were cultured at 25 °C to induce exogenous TDP-43 expression at a lower level. Two-day-old flies after eclosion were transferred to 28 °C for 4 h every day for continuous 4 days to induce TDP-43 overexpression or overexpression with corresponding siRNAs or cDNAs using the GMR-Gal4/tub-Gal80^ts^-driver.

### Antibodies

Commercial antibodies used in this study are listed in Table S2. A customized rabbit antibody against FUNDC1 was described in the previous study [11].

### Cell cultures and transfection

T-Rex tetracycline-inducible HEK293 cells stably expressing TDP-43 were described in our previous study [5]. FUNDC1 KO MEF cells were obtained from FUNDC1-deficient mice established in the previous study [25]. HEK293 cell line was from ATCC (Manassas, VA). SH-SY5Y cell line was purchased from the Cell Resource Center (Institute of Basic Medical Sciences, CAMS/PUMC, Beijing). All cells were cultured (37 °C, 5% CO_2_) in DMEM (Gibco, Waltham, MA), supplemented with 10% FBS (Gibco).

Cells were transfected with pcDNA4 TO plasmids expressing wild type or mutant of FUNDC1 with a myc-His tag using VigoFect (Vigorous Biotechnology, Beijing, China) or Lipofectamine^TM^ 3000 reagent (Invitrogen, Waltham, MA) following the manufacturer’s manual.

### Mito-Keima assay

Viral vector carrying mito-Keima was transfected into T-Rex inducible HEK293 cells stably expressing TDP-43. The cells were seeded into confocal dishes (NEST, Wuxi, China) and treated with tetracycline (1 μg/mL) to induce TDP-43 overexpression for 72 h. Mito-Keima fluorescence was captured using a Leica SP8 confocal microscope (Mannheim, Germany) via two sequential excitations (488 nm; 568 nm) using the same 620 nm emission. The fluorescent signal from 488 nm laser excitation was depicted in green, and the signal from 568 nm laser excitation was depicted in red.

### Transmission electron microscopy (TEM)

TEM was performed as published previously [5]. Briefly, fly heads were collected, fixed in 4% paraformaldehyde (Sinopharm Chemical Reagent Co., Ltd, Shanghai, China) and 2.5% glutaraldehyde (Sinopharm Chemical Reagent) overnight at 4 °C. The samples were sectioned on a Leica EM UC6/FC6 Ultramicrotome (Mannheim, Germany) and transferred to copper grids. EM imaging was carried out under a Tecnai^TM^ Spirit TEM (FEI, Hillsboro, OR) following counter staining using uranyl acetate and lead acetate.

### Biochemical fractionation experiments

One hundred fly heads were collected and transferred into a Glass-Teflon Dounce homogenizer containing cold isolation buffer (225 mM Mannitol, 75 mM Sucrose, 10 mM MOPS, 1 mM EDTA, 2.5 mg/mL BSA, 0.25×protease inhibitor cocktail, pH 7.2) and homogenized on ice for 20–30 strokes. Transfer 1/200th of the total volume of the homogenate to a new tube as the “Total lysates”. The remaining homogenate was centrifuged at 800 × *g* for 10 min at 4 °C, with supernatant saved for post-nuclear fraction (PNF). The PNF was centrifuged at 8000 × *g* for 10 min at 4 °C to enrich mitochondria, and the supernatant was transferred to a new tube as the “Cytosol fraction”. The mitochondrial pellets were washed twice with 1 mL wash buffer (225 mM Mannitol, 75 mM Sucrose, 10 mM KCl, 10 mM Tris-HCl, 5 mM KH_2_PO_4_, pH 7.4) at 8000 × *g* for 5 min at 4 °C, and the pellets were then lysed using RIPA buffer (150 mM NaCl, 50 mM Tris-HCl, pH 7.4, supplemented with 0.5% NP-40, 1 mM EDTA, 1 mM PMSF, 1× protease inhibitor cocktail). The protein concentrations of different cellular fractions were determined using the BCA assay (Pierce, Thermo Scientific, Rockford, USA), 2× loading buffer (20 mM Tris–HCl, pH 8.0, 100 mM DTT, 2% SDS, 20% Glycerol, and 0.016% Bromophenol Blue) was then added, and the samples were boiled and examined using SDS-PAGE followed by immunoblotting with corresponding antibodies.

Biochemical fractionation from cultured cells was similar to that for fly heads, except using different buffers. Isolation buffer: 225 mM Mannitol, 70 mM Sucrose, 20 mM HEPES, pH 7.2, and 1 mM EGTA, 0.25× protease inhibitor cocktail. Wash buffer: 250 mM Sucrose, 50 mM HEPES, pH 7.2, and 1 mM EGTA.

### Proteinase K protection assay

Mitochondrial fractions were prepared as described in “Biochemical fractionation experiments” and were treated with 100 μg/mL proteinase K in 50 μL of isolation buffer for 15 min at room temperature. Five mM PMSF was added to stop the reaction for 5 min at room temperature. Fifty μL of 2× loading buffer was added, and the samples were boiled and examined using SDS-PAGE followed by immunoblotting with corresponding antibodies.

### Co-immunoprecipitation (Co-IP)

Total cell lysates or mitochondrial fractions were lysed using RIPA buffer for co-IP with specific antibodies for 90 min at 4 °C, following incubation with protein A-agarose (Roche, Basel, Switzerland) for another 60 min at 4 °C. The protein A-agarose beads were washed using 1 mL RIPA buffer three times at 2400 × *g* for 1 min at 4 °C. Unless specified otherwise, 0.5% inputs and 20% immunoprecipitates were examined using SDS-PAGE followed by immunoblotting with corresponding antibodies.

To stabilize transient interaction, cells were incubated with PBS containing 0.25 mM dithiobis[succinimidylpropionate] (DSP, Sangon Biotech, Shanghai, China) for 30 min at 37 °C. To stop the reaction, 20 mM Tris-HCl, pH 7.8 was added at room temperature for 15 min. Mitochondria fractions prepared from DSP-treated cells were then used for Co-IP as described above.

### Purification of recombinant human TDP-43 from *E. coli* and HEK293 cells

A cDNA encoding human full-length TDP-43 protein was cloned into vector pET32M3C, expressed as a fusion protein carrying amino-terminal thioredoxin and 6×his-tag and purified from the *E. coli* Rosetta strain (Novagen, Darmstadt, Germany). Bacteria were grown at 37 °C in LB medium with 100 μg/ml ampicillin and induced with 0.5 mM isopropyl-D-thiogalactopyranoside (IPTG) for 18 h at 16 °C. The cells were harvested and suspended in buffer A (0.2 M NaCl, 25 mM Tris, pH 8.0, 20% glycerol, 2 mM β-mercaptoethanol) and French pressed on ice at 1200 bar for 4 times. The lysate was centrifuged, and the pellet was resuspended with NTA buffer (0.5 M NaCl, 25 mM Tris, pH 8.0, 20% glycerol) containing 8 M Urea and loaded onto a Ni-NTA column (GE Healthcare, Chicago, IL). The column was washed with NTA buffer containing 8 M Urea and then TDP-43 bound to the column was eluted with NTA buffer containing 0.25 M imidazole and 8 M Urea. The collected fraction was either flash-freeze or dialyzed against 20 mM Tris, pH 8.0, 0.2 M NaCl for the following experiment. Human TDP-43 protein with a myc-his-tag was purified using Ni-Sepharose (GE Healthcare) following Tet-induction (1 μg/mL tetracycline) of the inducible HEK293 cells for 36 h. Purified human TDP-43 was analyzed by SDS-PAGE followed by Coomassie Brilliant Blue staining or by immunoblotting with anti-TDP-43 or anti-myc antibody.

### Mitochondrial import assay

Mitochondria were purified according to “Biochemical fractionation experiments”. Mitochondria were suspended in 500 μL import buffer (250 mM Sucrose, 10 mM MOPS-KOH, pH 7.2, 80 mM KCl, 5 mM MgCl_2_, 3% BSA, 4 mM ATP, 4 mM NADH), and 10 μg purified TDP-43 was then added to start the import assay. Unless specified otherwise, the solution was incubated for 30 min at room temperature. The mitochondria were collected following centrifugation at 8000 × *g* for 5 min at 4 °C. The mitochondria pellets were washed twice with 1 ml wash buffer at 8000 × *g* for 5 min at 4 °C, followed by “Proteinase K protection assay”.

### siRNA transfection

The siRNA was from RIBOBIO (Guangzhou, China). Corresponding siRNA (siDNAJA2: 450 pmol; siHSPA8: 200 pmol every 24 h; siTOM70: 450 pmol) was incubated with 30 μL lipofectamine^TM^ 3000 reagent for 15 min at room temperature. The siRNA-lipid complex was then added to the HEK293 cells cultured in 10 cm dishes. Following the transfection (siDNAJA2: 72 h; siHSPA8: 48 h; siTOM70: 72 h), the cells were harvested and mitochondria were purified, followed by “Proteinase K protection assay” and immunoblotting analysis. In different groups, the dosage and transfection time for the negative control siRNA (NC) were the same as the siRNAs targeting corresponding genes. The target sequences of siRNAs were listed as follows: *DNAJA2*: 5’-GGAAGAAGGTGATTAAAGA-3’; *HSPA8*: 5’-GCTGGTCTCAATGTACTTA-3’; *TOM70*: 5’-GAGGCAGCATGTACATGCA-3’.

### RNA-seq data analysis

RNA-seq datasets of human frontal cortex samples were downloaded from GEO database, including those from control subjects (GSE100796 [41], GSE47966 [42], GSE68719 [43], GSE80655 [44]) and TDP-43 proteinopathy patients (GSE153960 [45]). Sratoolkit (v2.10.9) was used to extract FASTQ files. Following quality control using FastQC (v0.11.9), Trimmomatic (v0.39) [46] was used to remove adapters and trim low-quality reads and sequences. Clean reads were then mapped to the human reference transcriptome (Ensembl, GRCh38, release version 102) using Salmon (v1.4.0) [47]. Transcript-level expression was transformed to gene-level count value using tximport (v1.22.0) [48]. Gene expression was then normalized to TPM (transcript per million) based on count value and effective length [49]. Differential gene expression analysis was performed using DESeq2 (v1.34.0) [50]. Gene enrichment analyses were operated using Metascape [51]. Co-expression networks were constructed using MEGENA [24] with all protein-coding genes whose expression was detected at mean TPM ≥ 1 as input. Data sources are listed in Table S3.

### Statistical analyses

Quantification of immunoblotting, TEM, and fluorescence data was performed using ImageJ2 (v2.3.0). Data were collected in Excel (Microsoft) and analyzed using the R program (v3.3.3). Differences between different groups were analyzed using a one-sided Student’s *t*-test, One-way analysis of variance (ANOVA), or Two-way ANOVA followed by a post hoc test. For enumeration data, differences between different groups were analyzed using the Chi-square test or Fisher’s exact test, and Bonferroni correction was used for multiple comparisons. Measurement data are presented as mean ± standard error of the mean (SEM). Significance is indicated by asterisks: *P < 0.05; **P < 0.01; ***P < 0.001. The correlation coefficient was calculated using the Spearman method. Graphs were prepared using the R package “ggplot2” (v2.0.0) [52].

### Supplementary information

Reproducibility checklist

Supplementary information

Original Data File

## Data Availability

All data are available within the text and Supplementary Information.
