# Supplementary material for: Integration of FUNDC1-associated mitochondrial protein import and mitochondrial quality control contributes to TDP-43 degradation
Source: Cell Death Dis. 2023 Nov 11;14(11):735. doi: 10.1038/s41419-023-06261-6 (PMC10640645; doi:10.1038/s41419-023-06261-6)
Supplement: Supplementary file 2 — Supplementary information [file 41419_2023_6261_MOESM2_ESM.docx]

**Integration of FUNDC1-associated mitochondrial protein import and mitochondrial quality control contributes to TDP-43 degradation**

Jinfa Ma^1,2^, Lei Liu^3^, Lu Song^1^, Jianghong Liu^1^, Lingyao Yang^1,2^, Quan Chen^4^, Jane Y. Wu^5,*^, Li Zhu^1,2,*^

^1^State Key Laboratory of Brain and Cognitive Science, Institute of Biophysics, Chinese Academy of Sciences, Beijing 100101, China; ^2^University of Chinese Academy of Sciences, Beijing 100049, China; ^3^State Key Laboratory of Membrane Biology, Institute of Zoology, Chinese Academy of Sciences, Beijing 100101, China; ^4^Interdisciplinary Center of Cell Response, State Key Laboratory of Medicinal Chemical Biology, College of Life Sciences, Nankai University, Tianjin 300071, China; ^5^Department of Neurology, Center for Genetic Medicine, Lurie Cancer Center, Northwestern University Feinberg School of Medicine, Chicago, Illinois 60611, USA.

*To whom correspondence may be addressed. Email: [jane-wu@northwestern.edu](mailto:jane-wu@northwestern.edu) or [zhuli@ibp.ac.cn](mailto:zhuli@ibp.ac.cn).

**Supplementary tables**

Table S1 Information of fly stocks.

| Fly stock | Source |
| --- | --- |
| GMR-Gal4; tub-Gal80^ts^ | Wang et al. (2019) |
| GMR-Gal4/UAS-RFP; tub-Gal80^ts^ | Wang et al. (2019) |
| GMR-Gal4, UAS-TDP-43-RFP-HA/Cyo; tub-Gal80^ts^ | Wang et al. (2019) |
| y v; attP40, y+ | Tsinghua Fly Center |
| UAS-siHsc70-3 | Tsinghua Fly Center |
| UAS-siHsc70-4 | Tsinghua Fly Center |
| UAS-siHsc70-5 | Tsinghua Fly Center |
| UAS-siCG567 | Tao Wang’s Lab, NIBS |
| UAS-CG5676orf-HA | Tao Wang’s Lab, NIBS |
| UAS-Hsc70-3orf-HA | Tao Wang’s Lab, NIBS |
| UAS-Hsc70-5orf | Tao Wang’s Lab, NIBS |

Table S2 Information of antibodies.

| Antibody | Source | Identifier |
| --- | --- | --- |
| Rabbit polyclonal anti-TDP-43 | ProteinTech | Cat# 10782-2-AP |
| Rabbit polyclonal anti-ATP5B | ProteinTech | Cat# 17247-1-AP |
| Rabbit polyclonal anti-Histone3 | ProteinTech | Cat# 17168-1-AP |
| Rabbit polyclonal anti-α-tubulin | ProteinTech | Cat# 11224-1-AP |
| Rabbit polyclonal anti-β-tubulin | ProteinTech | Cat# 10094-1-AP |
| Rabbit polyclonal anti-TOM70 | ProteinTech | Cat# 14528-1-AP |
| Rabbit polyclonal anti-TOM40 | ProteinTech | Cat# 18409-1-AP |
| Rabbit polyclonal anti-TOM20 | ProteinTech | Cat# 11802-1-AP |
| Rabbit polyclonal anti-LONP1 | ProteinTech | Cat# 15440-1-AP |
| Rabbit polyclonal anti-HSPA8 | ProteinTech | Cat# 10654-1-AP |
| Rabbit polyclonal anti-HSPA9 | ProteinTech | Cat# 14887-1-AP |
| Rabbit polyclonal anti-DNAJA2 | ProteinTech | Cat# 12236-1-AP |
| Rabbit polyclonal anti-LC3 | ProteinTech | Cat# 14600-1-AP |
| Rabbit polyclonal anti-FUNDC1 | Cell Signaling | Cat# 49240 |
| Mouse monoclonal anti-TDP-43 | ProteinTech | Cat# 60019-2-Ig |
| Mouse monoclonal anti-GAPDH | ProteinTech | Cat# 60004-1-Ig |
| Mouse monoclonal anti-TIM23 | Transduction Laboratories | Cat# T85720 |
| Mouse monoclonal anti-HA | CoWin Biosciences | Cat# CW0092A |
| Mouse monoclonal anti-myc | CoWin Biosciences | Cat# CW0088 |
| Anti-rabbit IgG, HRP | GE Healthcare | Cat# NA934V |
| Anti-mouse IgG, HRP | GE Healthcare | Cat# NA931V |

Supplementary Table S3 Public RNA-seq data sources.

| Public RNA-seq data | Source |
| --- | --- |
| GSE153960 | https://www.ncbi.nlm.nih.gov/geo/query/acc.cgi?acc=GSE153960 |
| GSE47966 | https://www.ncbi.nlm.nih.gov/geo/query/acc.cgi?acc=GSE47966 |
| GSE68719 | https://www.ncbi.nlm.nih.gov/geo/query/acc.cgi?acc=GSE68719 |
| GSE80655 | https://www.ncbi.nlm.nih.gov/geo/query/acc.cgi?acc=GSE80655 |
| GSE100796 | https://www.ncbi.nlm.nih.gov/geo/query/acc.cgi?acc=GSE100796 |
| GTEx | https://storage.googleapis.com/gtex_analysis_v8/rna_seq_data/GTEx_Analysis_2017-06-05_v8_RNASeQCv1.1.9_gene_tpm.gct.gz |
| Allen Brain | https://portal.brain-map.org/atlases-and-data/rnaseq |

**Supplementary figures**


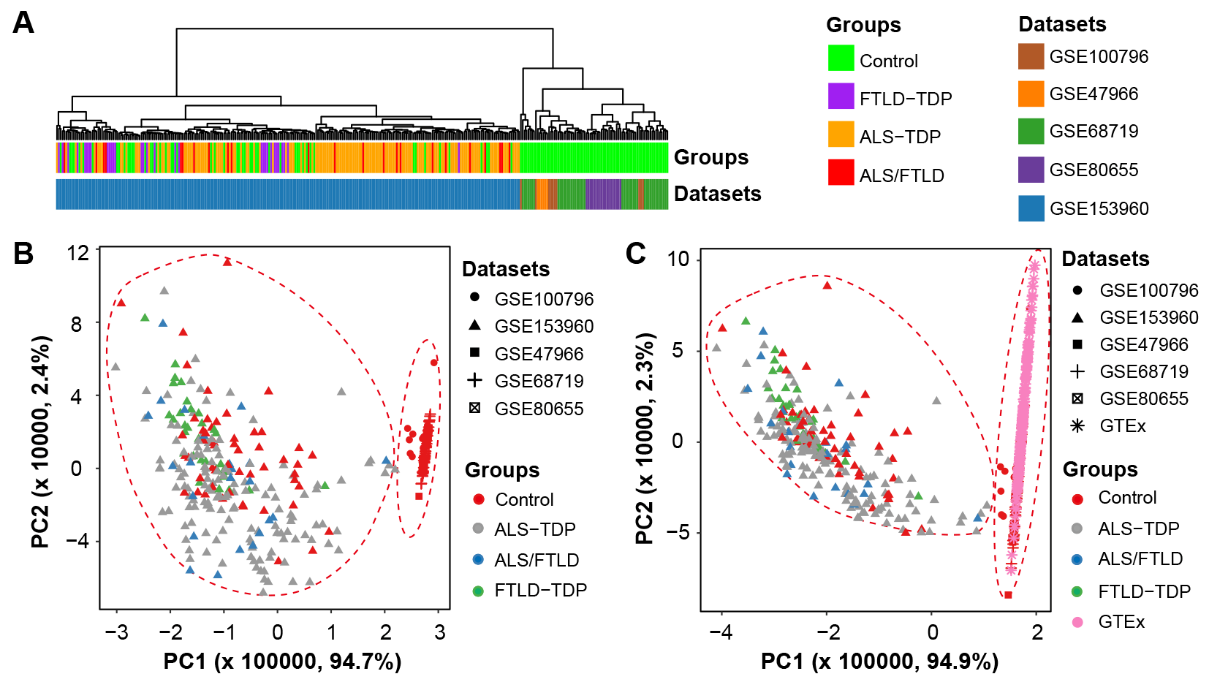


**Fig. S1 Quality control of RNA-seq datasets from control and TDP-43 proteinopathy samples.** Raw RNA-seq data from frontal cortex samples of the TDP-43 proteinopathy group (ALS-TDP, ALS/FTLD and FTLD-TDP) in GSE153960 and of control in GSE47966, GSE68719, GSE80655, GSE100796 and GSE153960 were downloaded. Following quality control, transcript expression was quantified using Salmon. **A.** Hierarchical clustering of control and TDP-43 proteinopathy samples. **B.** Principal component analysis (PCA) of control and TDP-43 proteinopathy samples. **C.** PCA of control and TDP-43 proteinopathy samples. Expression data of the frontal cortex in the GTEx datasets were included to perform PCA. Results in **A**-**C** show that “control” samples in the GSE153960 dataset were clustered together with patient samples in the TDP-43 proteinopathy group. Therefore, normal control samples in GSE47966, GSE68719, GSE80655, and GSE100796 were included as controls.


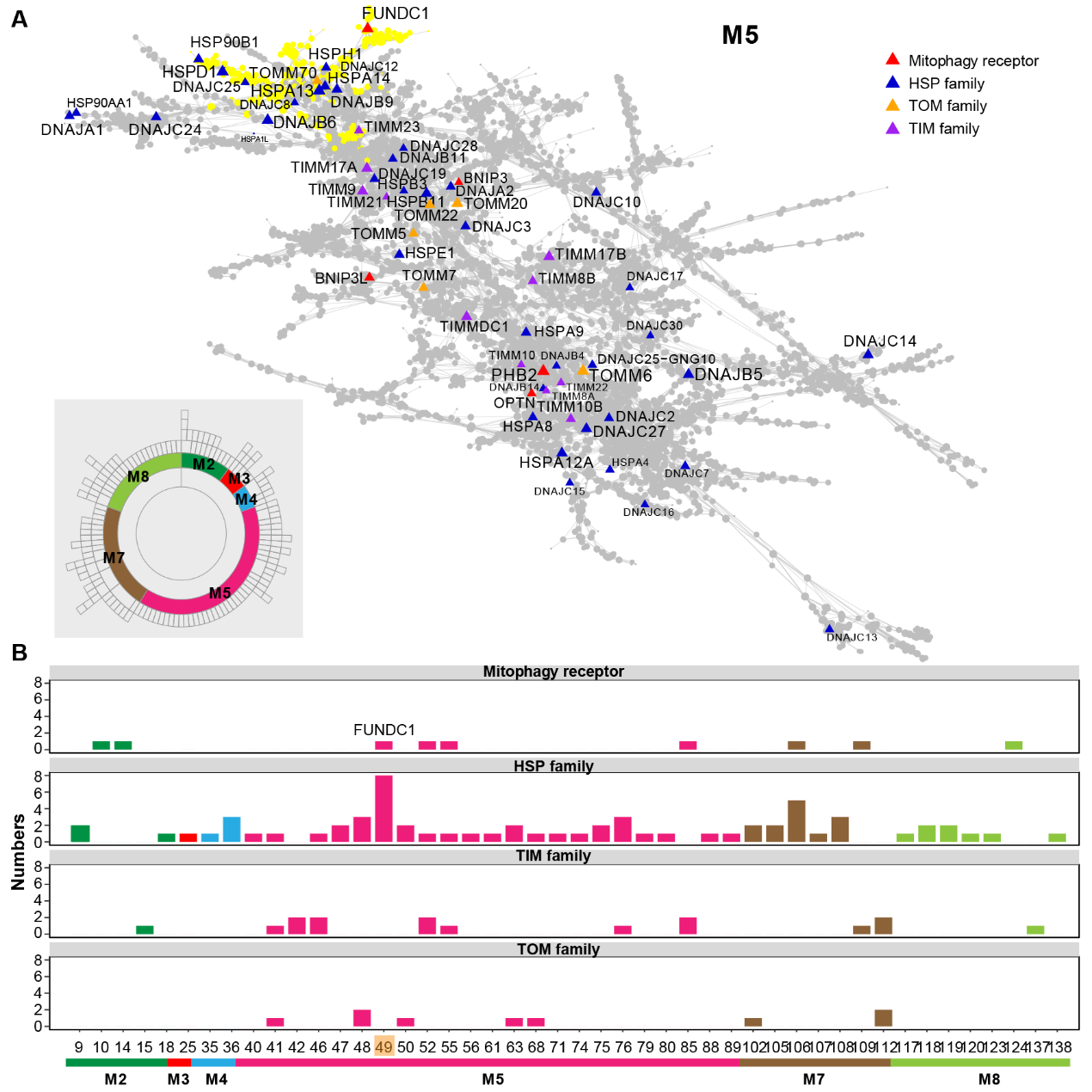


**Fig. S2 Co-expression network analysis of modules containing mitophagy receptor genes in control samples. A.** The hierarchical structure of the entire co-expression network is shown at the bottom left. Detailed Analyses show that M5, the largest module, contains *FUNDC1* and many members of HSP, TIM and TOM gene families. Yellow nodes are those of *FUNDC1*-containing child module M49. **B.** Gene numbers and distribution of members of mitophagy receptor, HSP, TIM and TOM families in child modules of M2, M3, M4, M5, M7 and M8. FUNDC1-containing child module M49 contains more HSP family members than any other child modules.


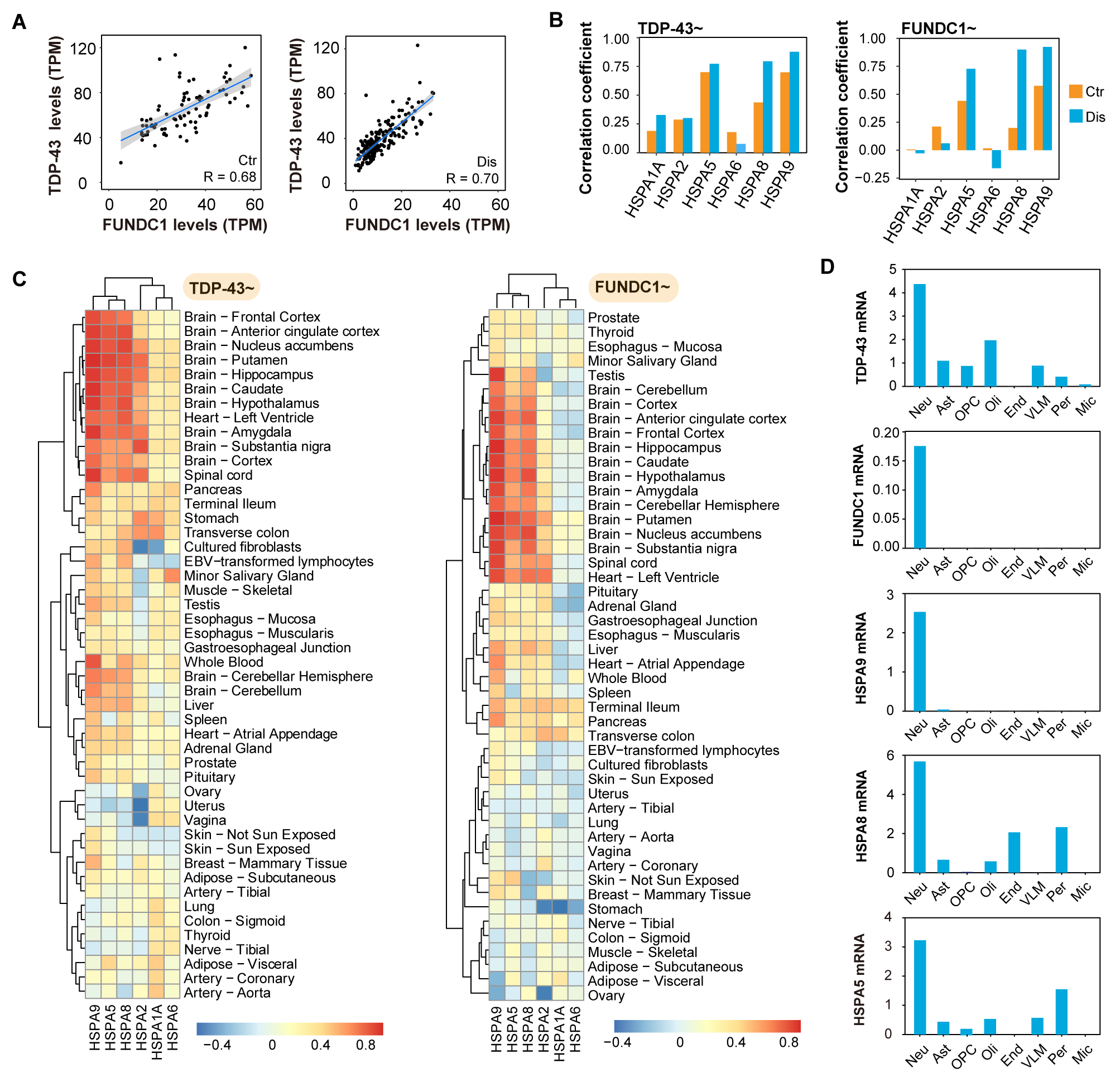


**Fig. S3 *TDP-43* and *FUNDC1* are co-expressed with *HSPA8/A9/A5*. A.** *TDP43* expression is positively correlated with that of *FUNDC1* in control (Ctr) and TDP-43 proteinopathy (Dis) samples. Blue lines describe the linear regression, and grey shading depicts 95% confidence intervals. Corresponding Spearman correlation coefficient R values were shown. **B.** Correlation of expression of *TDP-43* or *FUNDC1* with that of *HSPA1A, A2, A6, A8* and *A9* in control (Ctr) and TDP-43 proteinopathy (Dis) samples. **C.** Correlation of expression of *TDP-43* or *FUNDC1* with that of *HSPA1A, A2, A6, A8* and *A9* in different tissues in GTEx RNA-seq dataset. **D.** Gene expression data in different brain cell types in Allen Brain RNA-seq dataset. **Neu**: neurons; **Ast**: astrocytes; **OPC**: oligodendrocyte progenitor cells; **Oli**: Oligodendrocyte; **End**: Endothelial cells; **VLM**: vascular leptomeningeal cells; **Per**: Pericyte; **Mic**: Microglia.


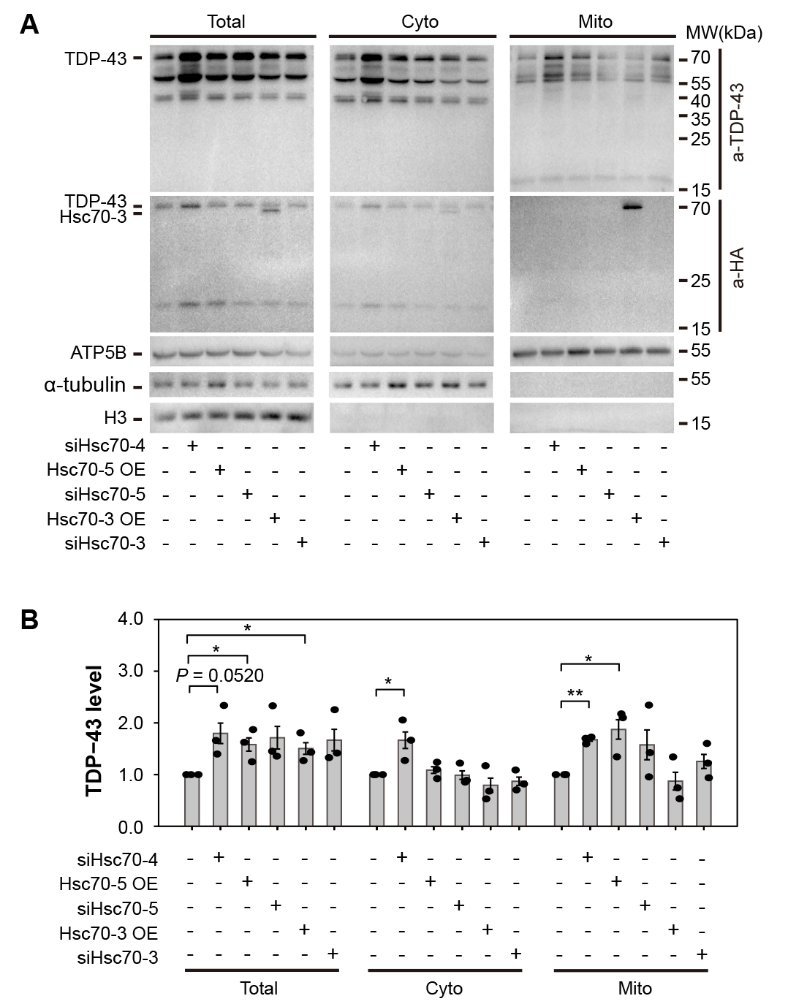


**Fig. S4 HSPA8 and HSPA9 affect mitochondrial translocation of TDP-43 in flies. A-B.** Immunoblotting (**A**) and quantification (**B**) of the TDP-43 protein levels of male files in different groups. Data are presented as mean ± SEM and analyzed using a Student’s *t*-test (n = 3 independent experiments; ** *P* < 0.01, * *P* < 0.05). Hsc70-3, fly homolog of human HAPA5; Hsc70-4, fly homolog of human HAPA8; Hsc70-5, fly homolog of human HAPA9. All the files were under TDP-43 overexpressing background, fly genotypes: **siHsc70-4**: GMR-Gal4/tub-Gal80^ts^/UAS-TDP-43-RFP-HA/UAS-siHsc70-4; **Hsc70-5** **OE**: GMR-Gal4/tub-Gal80^ts^/UAS-TDP-43-RFP-HA/UAS-orfHsc70-5; **siHsc70-5**: GMR-Gal4/tub-Gal80^ts^/UAS-TDP-43-RFP-HA/UAS-siHsc70-5; **Hsc70-3** **OE**: GMR-Gal4/tub-Gal80^ts^/UAS-TDP-43-RFP-HA/UAS-orfHsc70-3-HA; **siHsc70-3**: GMR-Gal4/tub-Gal80^ts^/UAS-TDP-43-RFP-HA/UAS-siHsc70-3.


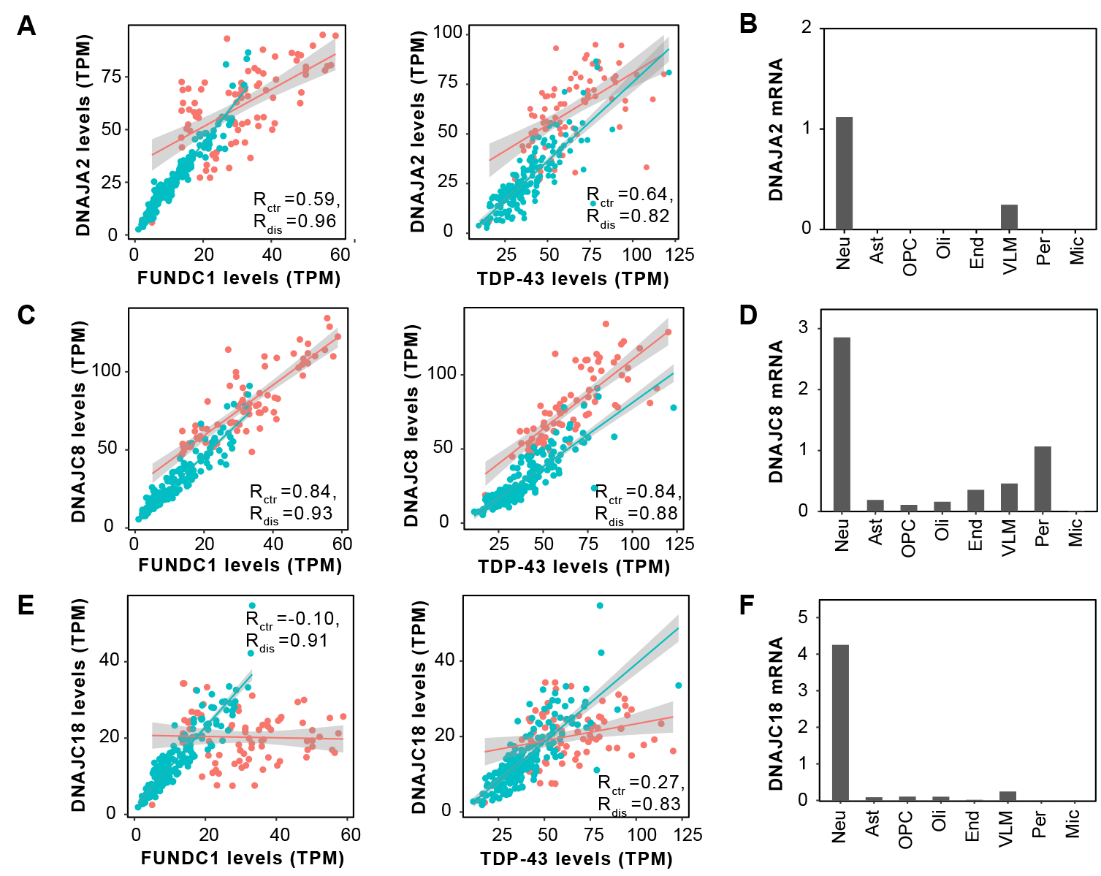


**Fig. S5 Co-expression of *DNAJA2*, *DNAJC8* and *DNAJC18* with *FUNDC1* and *TDP-43*. A, C, E.** Correlation coefficients of *DNAJA2* (**A**), *DNAJC8* (**C**), *DNAJC18* (**E**) with *FUNDC1* or *TDP-43* in control and TDP-43 proteinopathy samples. R: Spearman correlation coefficient; Ctr: control; Dis: TDP-43 proteinopathy. **B, D, F.** RNA expression of *DNAJA2* (**B**), *DNAJC8* (**D**), *DNAJC18* (**F**) in different cell types. The RNA expression data of different cell types are from Allan Brain. **Neu**: neurons; **Ast**: astrocytes; **OPC**: oligodendrocyte progenitor cells; **Oli**: Oligodendrocyte; **End**: Endothelial cells; **VLM**: vascular leptomeningeal cells; **Per**: Pericyte; **Mic**: Microglia.


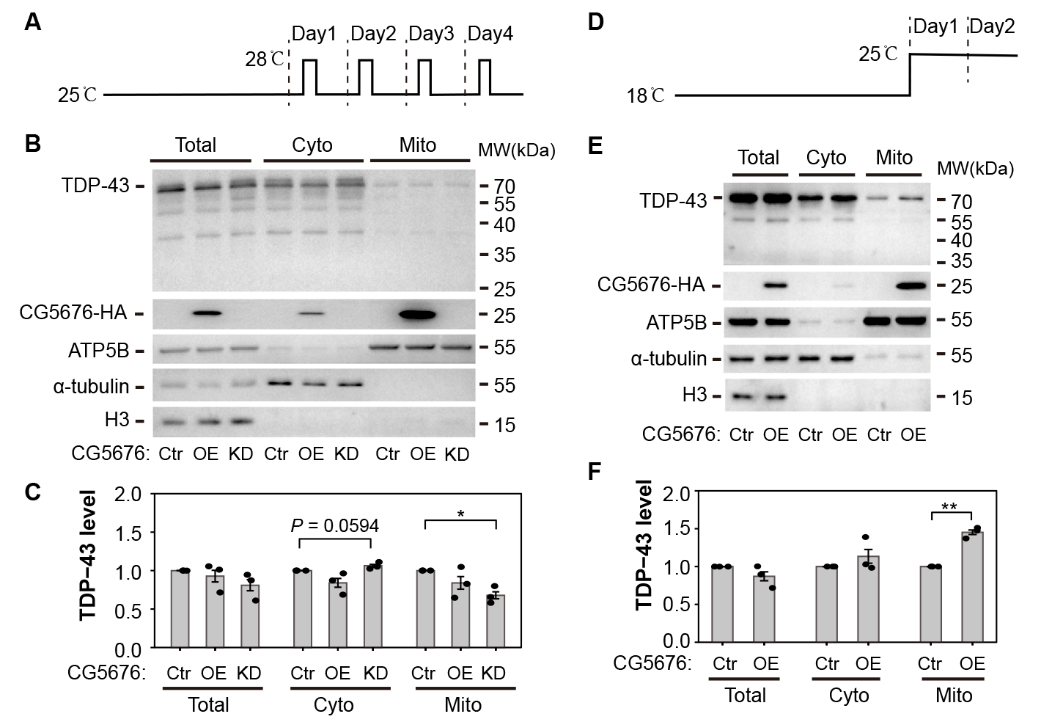


**Fig. S6 Altering fly FUNDC1 (CG5676) expression affects cytosolic and mitochondrial TDP-43 level in a condition dependent manner. A.** Diagram of the standard fly heat shock protocol in this study. **B-C.** Immunoblotting (**B**) and quantification (**C**) of total, cytosolic and mitochondrial TDP-43 after altering fly FUNDC1 (CG5676) expression. Ctr: control, OE: overexpression; KD: knocking down. **D.** Diagram of a moderate heat shock protocol. **E-F.** Immunoblotting (**E**) and quantification (**F**) of total, cytosolic and mitochondrial TDP-43 under moderate heat shock protocol after overexpressing fly FUNDC1. The full-length TDP-RFP-HA bands (~70kDa) were used for quantification in **C** and **F**. Data in **C** and **F** are presented as mean ± SEM and analyzed by a Student’s *t*-test (n = 3 independent experiments; ** *P* < 0.01, * *P* < 0.05). Male flies were used in sub-cellular TDP-43 protein analysis. All the files were under TDP-43 overexpressing background, fly genotypes: **Ctr**: GMR-Gal4/tub-Gal80^ts^/UAS-TDP-43-RFP-HA/attp40; **OE**: GMR-Gal4/tub-Gal80^ts^/UAS-TDP-43-RFP-HA/UAS-orfCG5676-HA; **KD**: GMR-Gal4/tub-Gal80^ts^/UAS-TDP-43-RFP-HA/UAS-siCG5676.


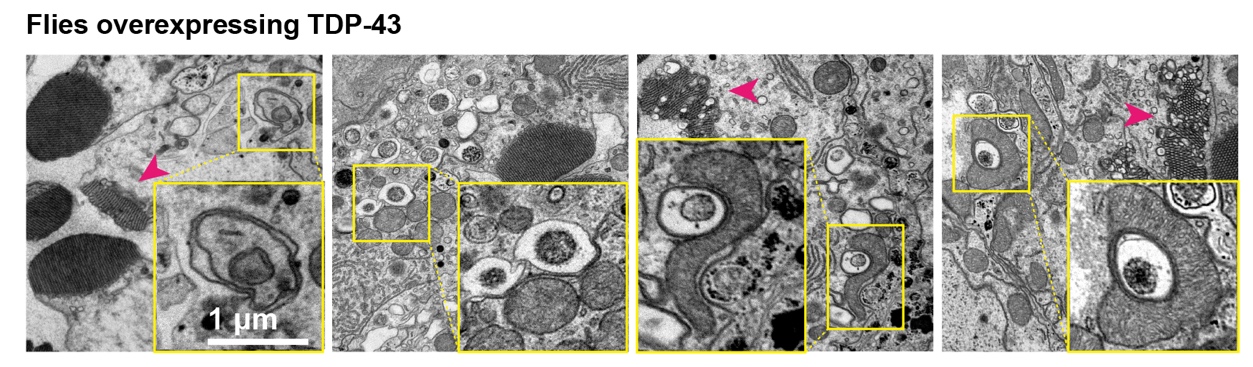


**Fig. S7 Overexpressing TDP-43 activates mitophagy in flies.** TEM imaging of mitophagy in male flies overexpressing TDP-43. Left two panels show mitochondria located within autophagosomes, and right two panels show mitochondria in contact with or fused with autophagosome, and mitochondria containing vacuole. Claret arrows mark the degenerated rhabdomeres. Scale bar: 1 μm.


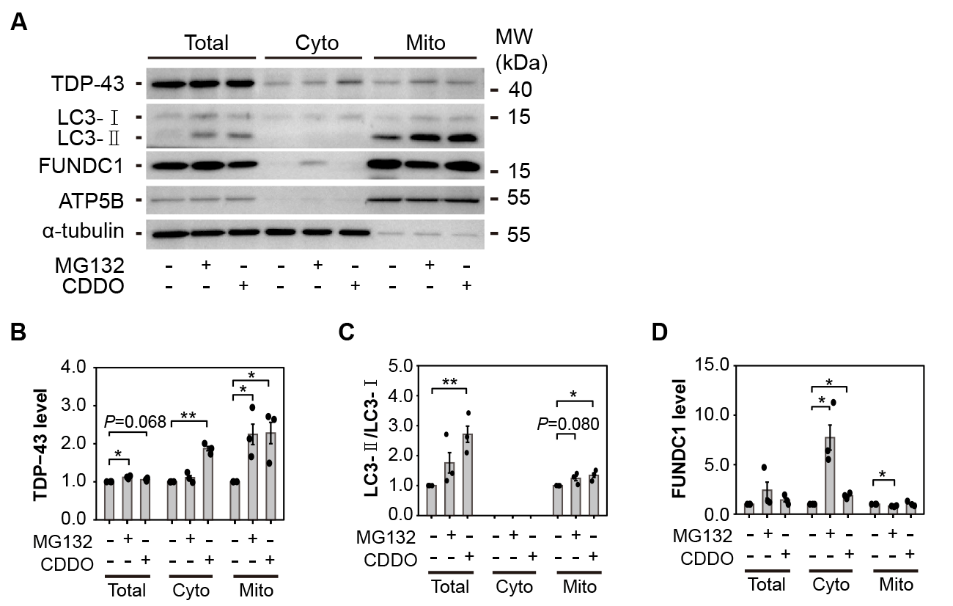


**Fig. S8 Inhibiting ubiquitin-proteasome system or LONP1 increases mitochondrial TDP-43 level and LC3-II/LC3-I ratio**. Immunoblotting (**A**) and quantification (**B-D**) of TDP-43, LC3 and FUNDC1 after inhibiting proteasome by MG132 (10 μM) or LONP1 by CDDO (5 μM) in HEK293 cells for 48 hours. Data are presented as mean ± SEM and analyzed using a Student’s *t*-test (n = 3 independent experiments; ** *P* < 0.01, * *P* < 0.05).


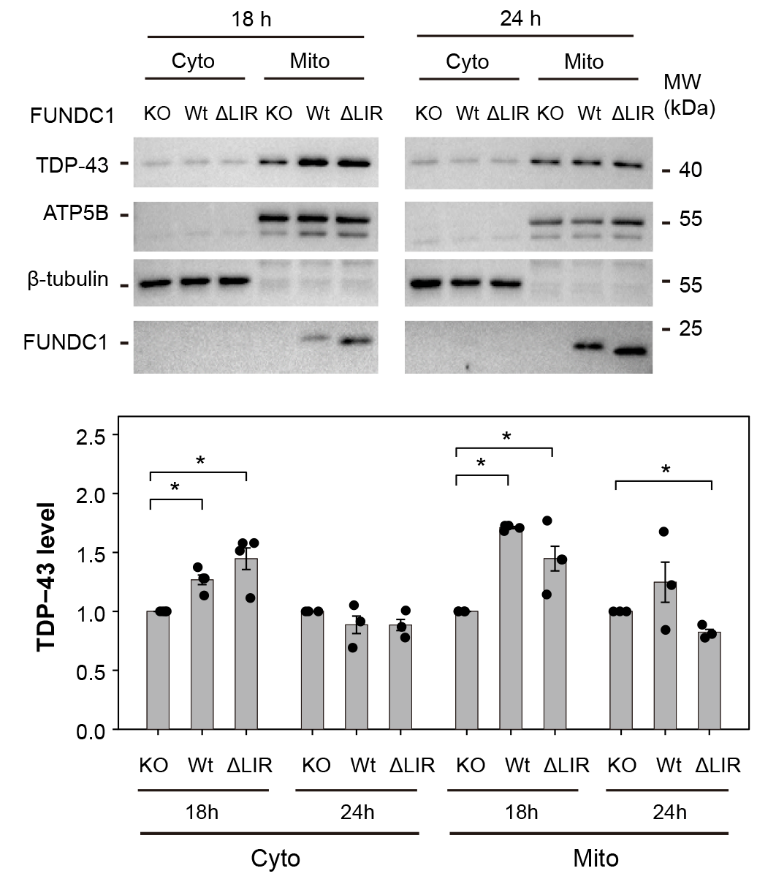


**Fig. S9 Overexpressing FUNDC1 dynamically regulates cytosolic and mitochondrial TDP-43 levels in MEF cells.** FUNDC1 knockout (KO) MEF cells were transfected with wildtype (Wt) and LIR deletion mutant (ΔLIR) of FUNDC1 for 18 h and 24 h, respectively. Cytosolic and mitochondrial TDP-43 levels were detected by immunoblotting. Data are presented as mean ± SEM and analyzed by a Student’s *t*-test (* *P* < 0.05).
