# Supplementary material for: Integration of FUNDC1-associated mitochondrial protein import and mitochondrial quality control contributes to TDP-43 degradation
Source: Cell Death Dis. 2023 Nov 11;14(11):735. doi: 10.1038/s41419-023-06261-6 (PMC10640645; doi:10.1038/s41419-023-06261-6)
Supplement: Supplementary file 3 — Original Data File [file 41419_2023_6261_MOESM3_ESM.docx]

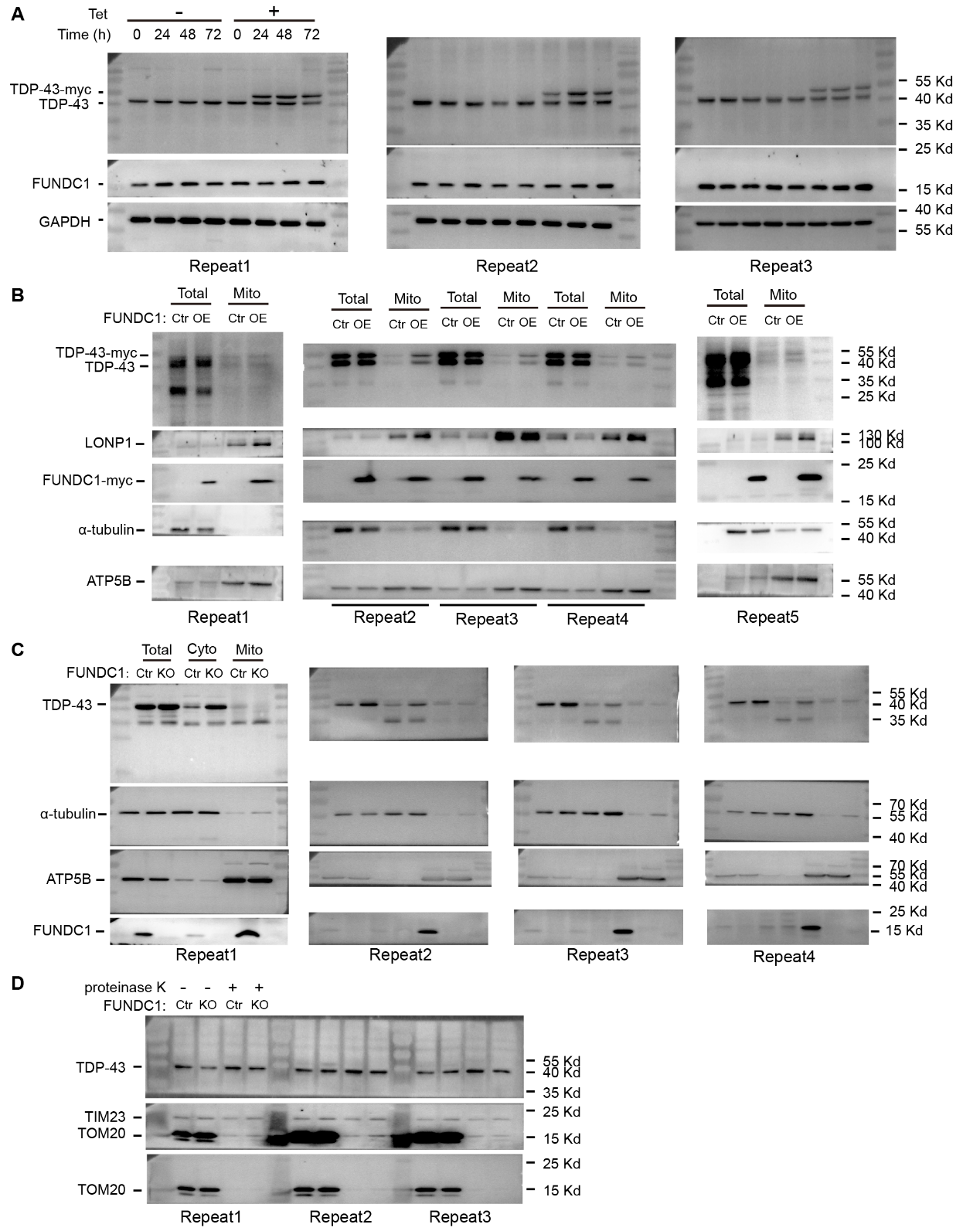


**Source data 1: FUNDC1 promotes mitochondrial translocation of TDP-43**. **A, B, C, D** show the source data of Fig 2A, 2B (repeat 1-4 were used to quantify the TDP-43 levels), 2C, 2D.


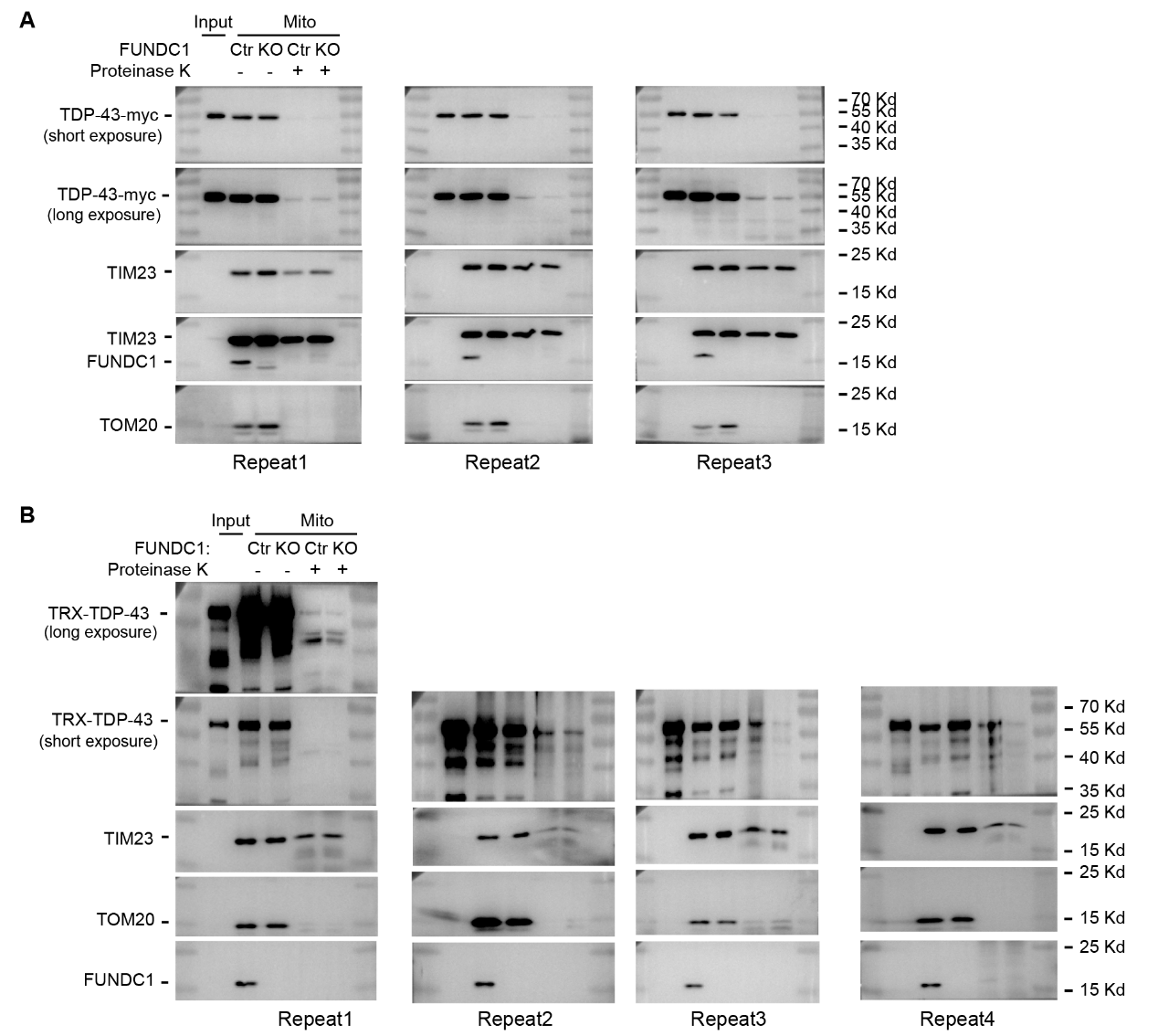


**Source data 2: FUNDC1 promotes mitochondrial TDP-43 import in vitro mitochondrial protein import assay**. **A, B** show the source data of Fig 2E, 2F.


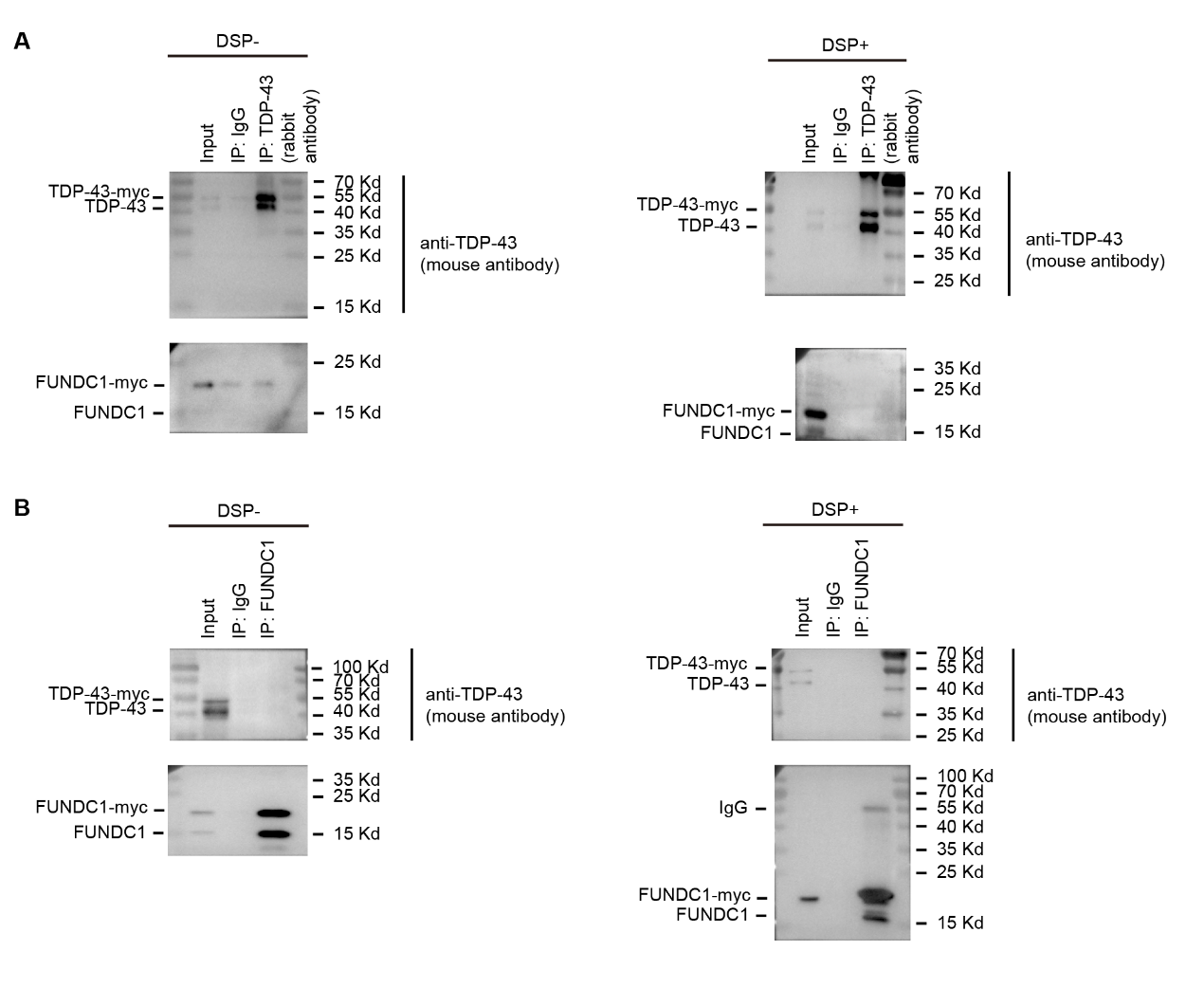


**Source data 3: No direct interaction is detected between FUNDC1 and TDP-43**. **A, B** show the source data of Fig 2H, 2I.


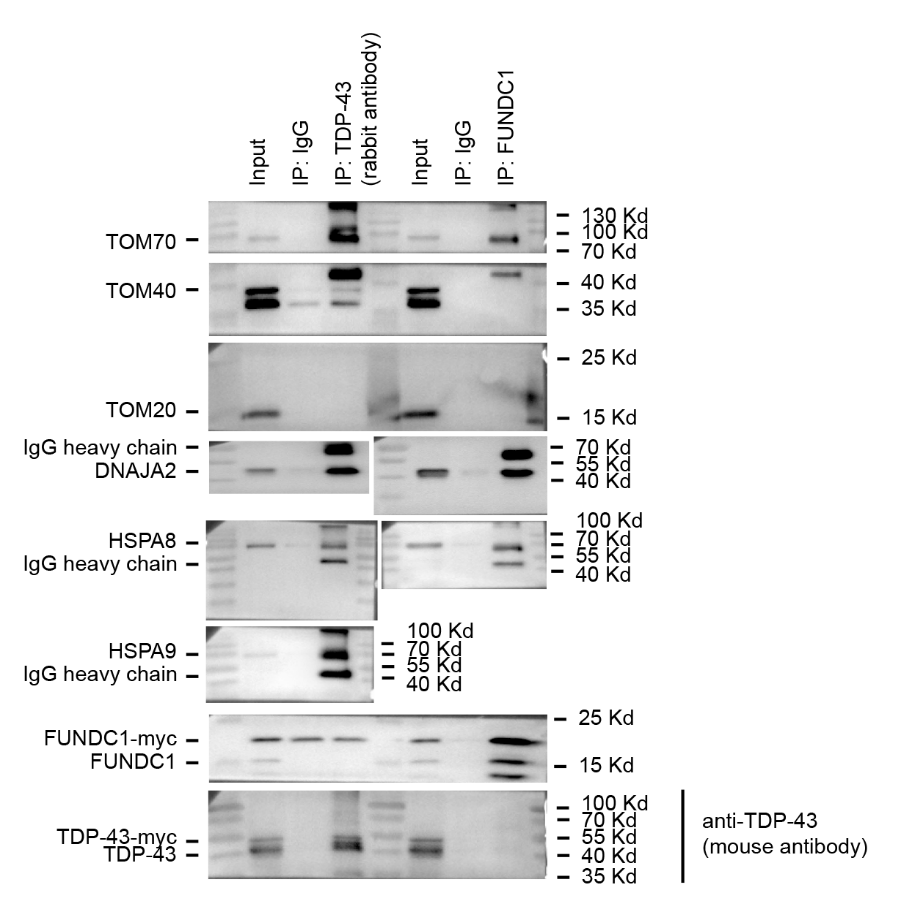


**Source data 4: TDP-43 and FUNDC1 interact with HSPA8/TOM70/DNAJA2**. The source data is of Fig 3A and Fig 3B.


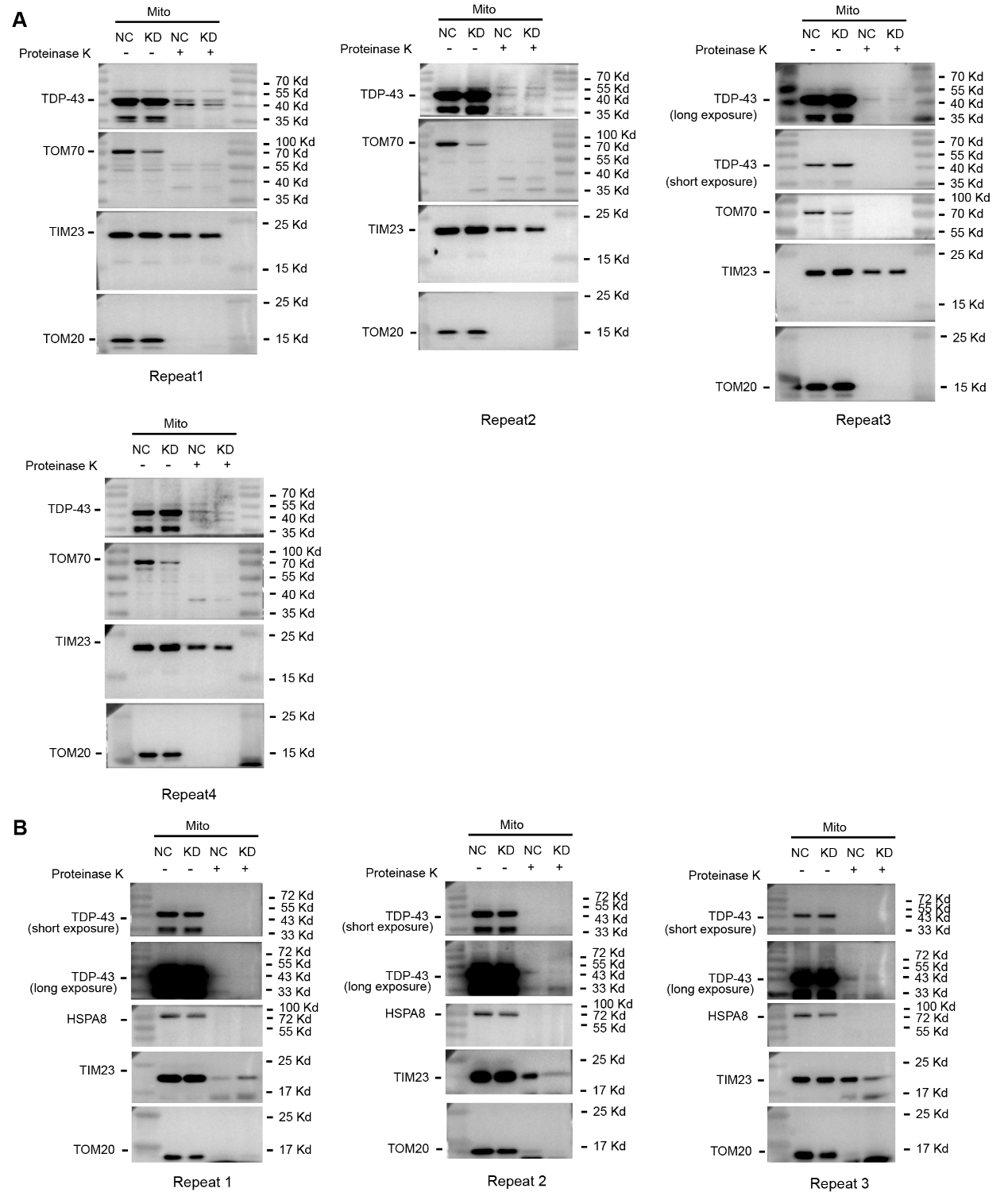


**Source data 5: Knocking down TOM70 or HSPA8 decreases mitochondrial TDP-43 levels**. **A, B** show the source data of Fig 3C and Fig 3D.


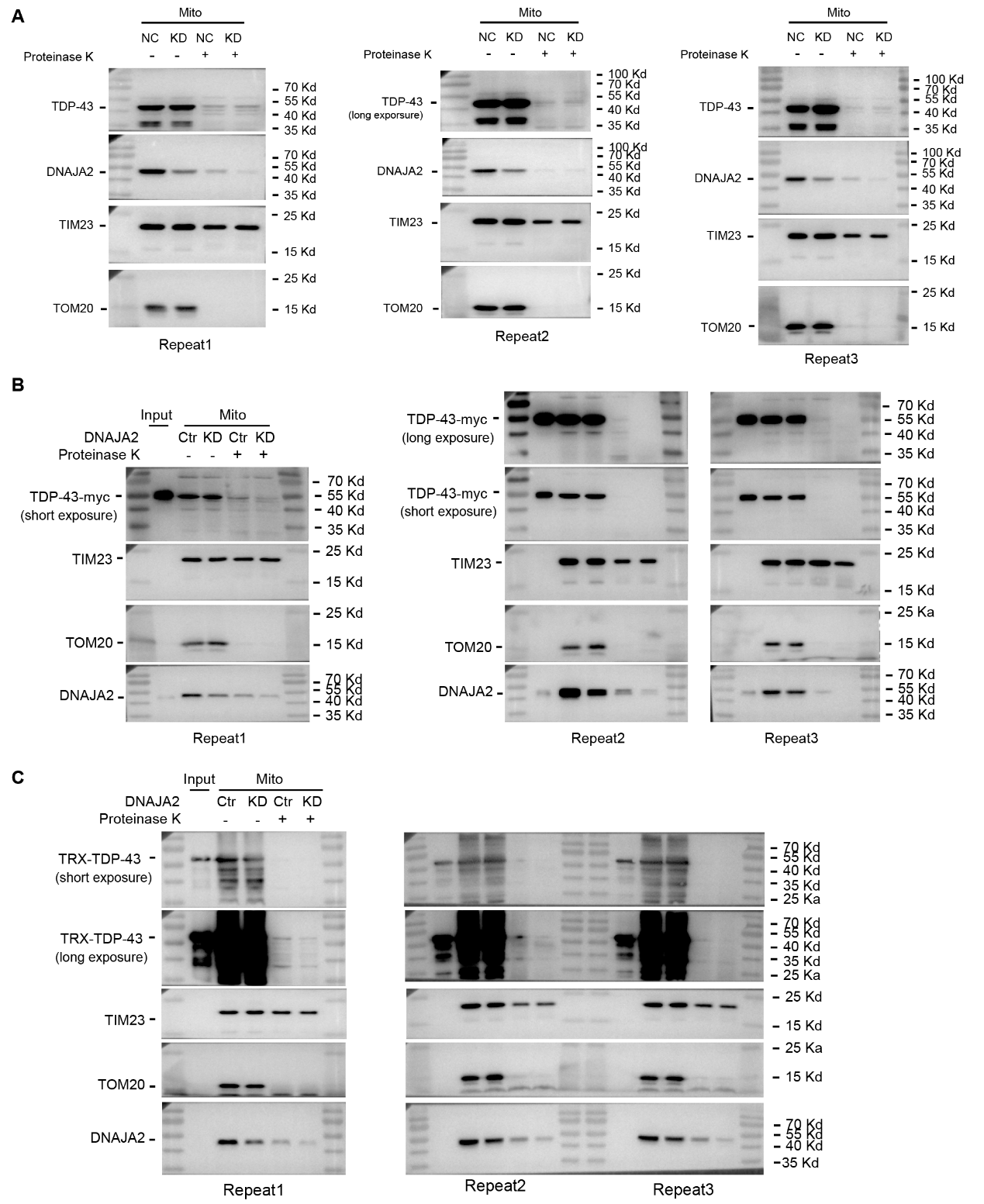


**Source data 6: Knocking down DNAJA2 decreases mitochondrial TDP-43 levels in vitro but not in vivo**. **A, B, C** show the source data of Fig 3E, 3F, 3G.


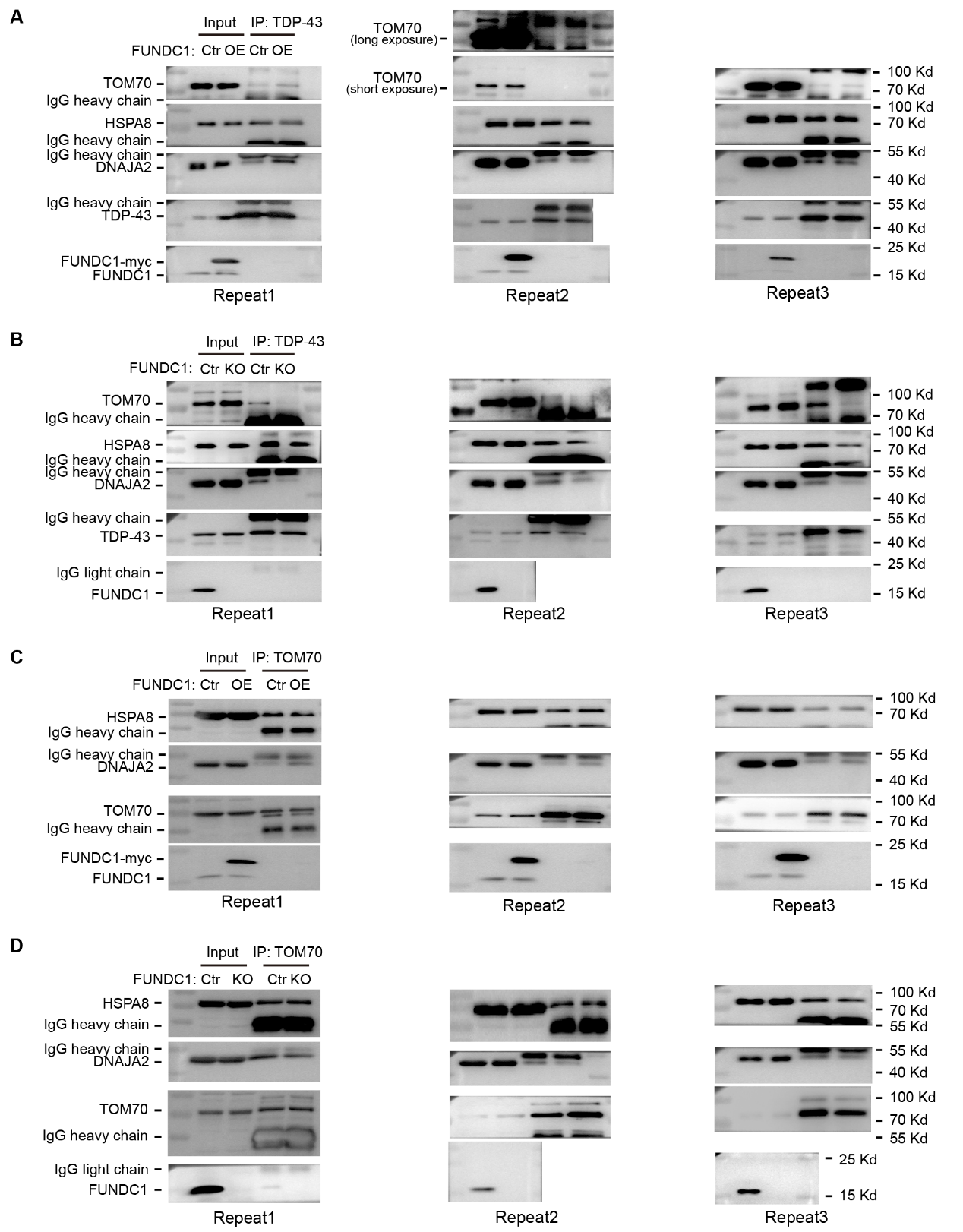


**Source data 7: FUNDC1 affects the interactions of proteins that are associated with mitochondrial protein import**. **A, B, C, D** show the source data of Fig 4A, 4B, 4C, 4D.


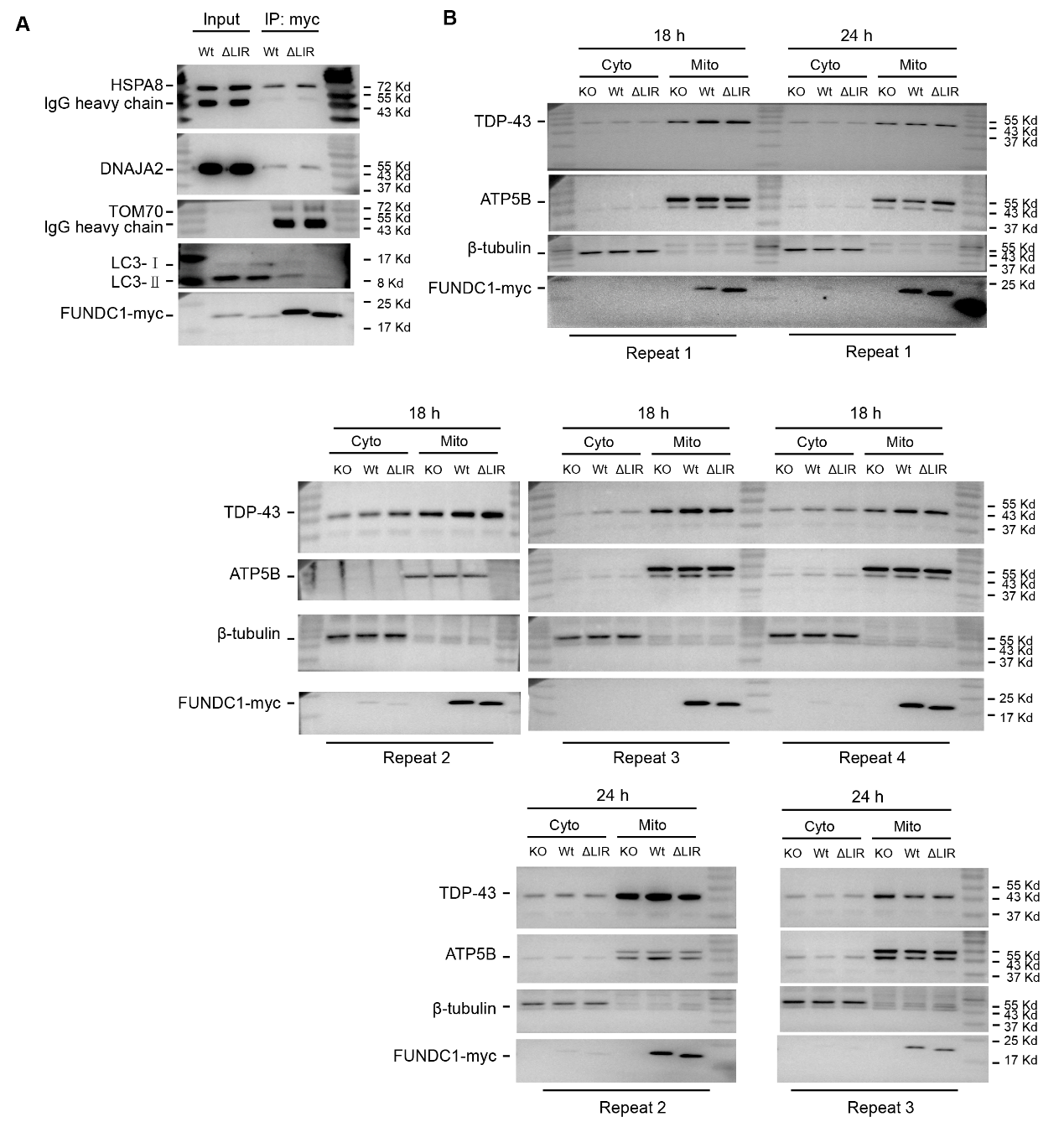


**Source data 8: FUNDC1 promotes mitochondrial translocation of TDP-43 independent on LIR motif**. **A, B, C** show the source data of Fig 4E, 4F and Fig S9.


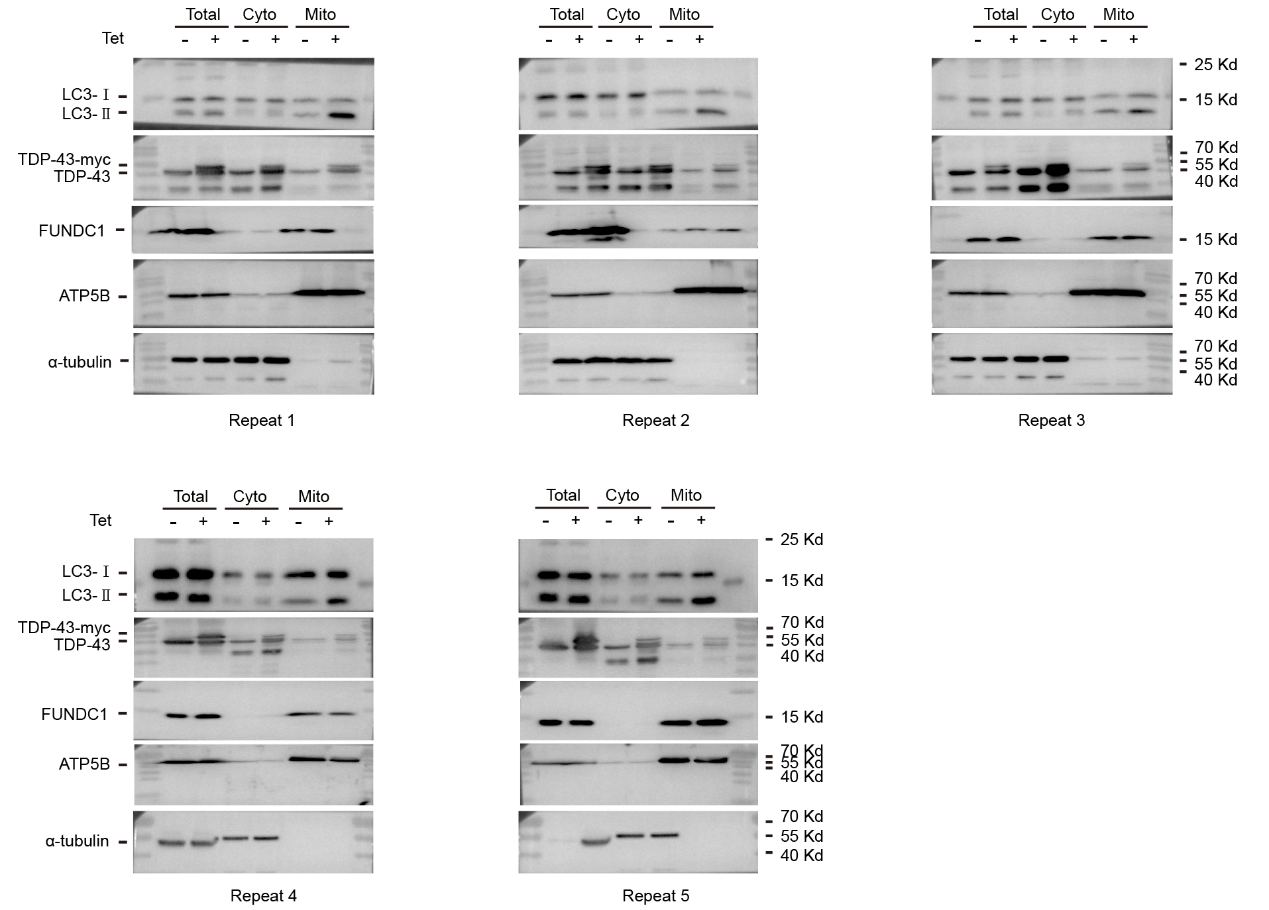


**Source data 9: Overexpressing TDP-43 increases mitochondrial FUNDC1 level and LC3-Ⅱ**/ **LC3-Ⅰ ratio**. The source data is of Fig 6C. Repeats 1-4 were used to quantify FUNDC1 and LC3 level.


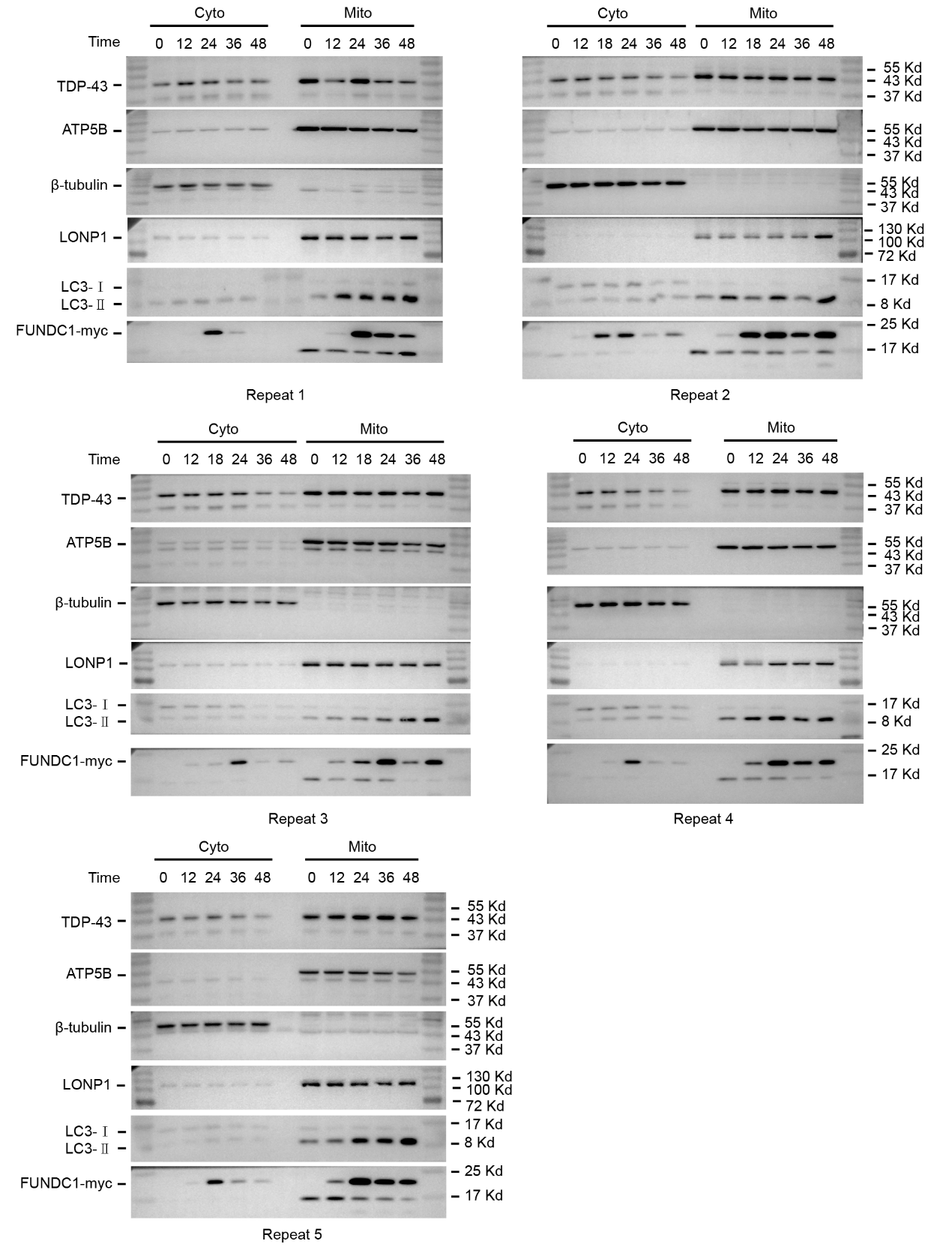


**Source data 10: Overexpressing FUNDC1 decreases cytosolic TDP-43 level.** The source data is of Fig 6F.


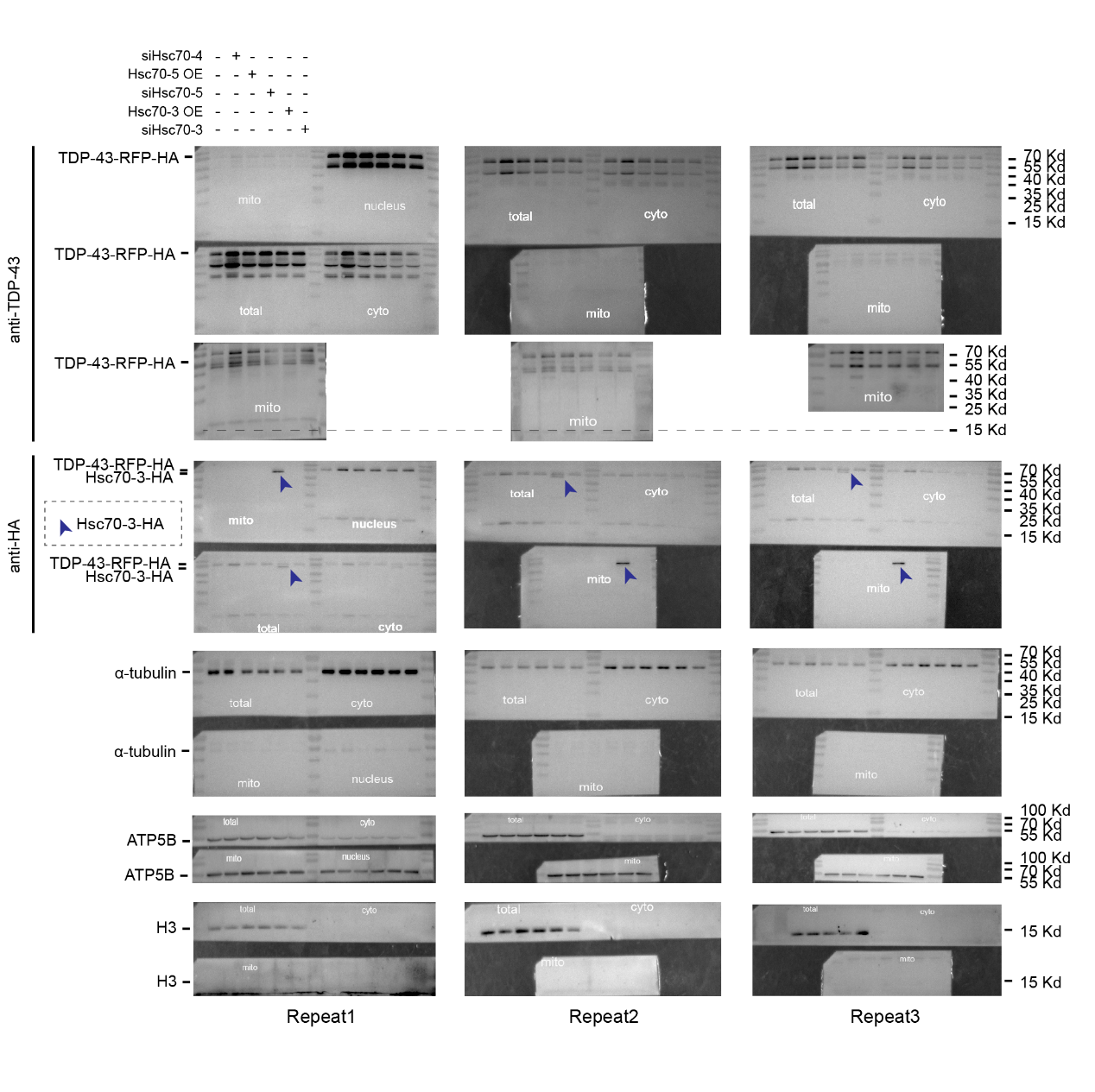


**Source data 11: HSPA8/A9 affects the mitochondrial TDP-43 levels**. The source data is of Fig S4.


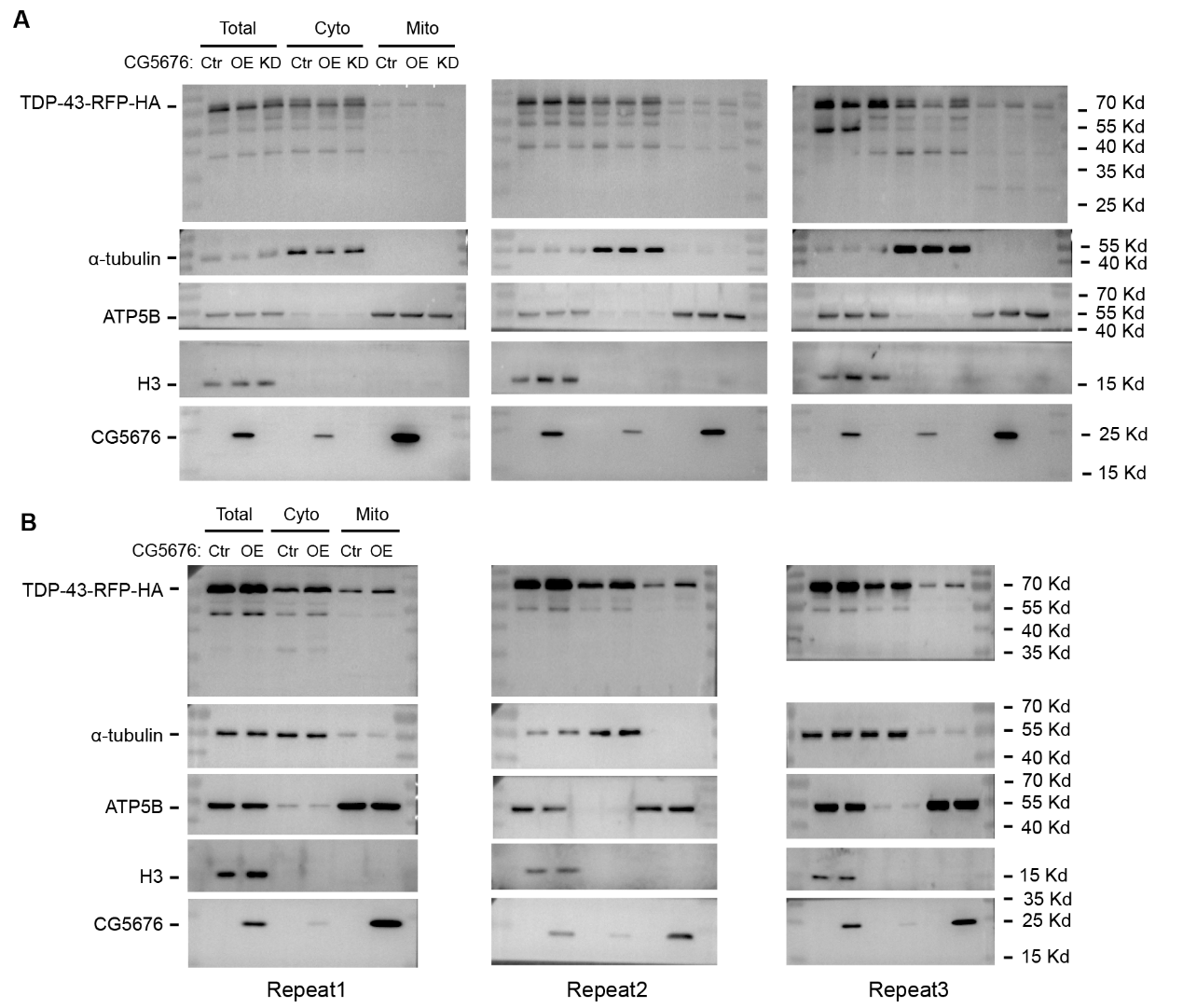


**Source data 12: Altering fly FUNDC1 (CG5676) affects sub-cellular distribution of TDP-43**. **A, B** show the source data of Fig S6B, S6E.


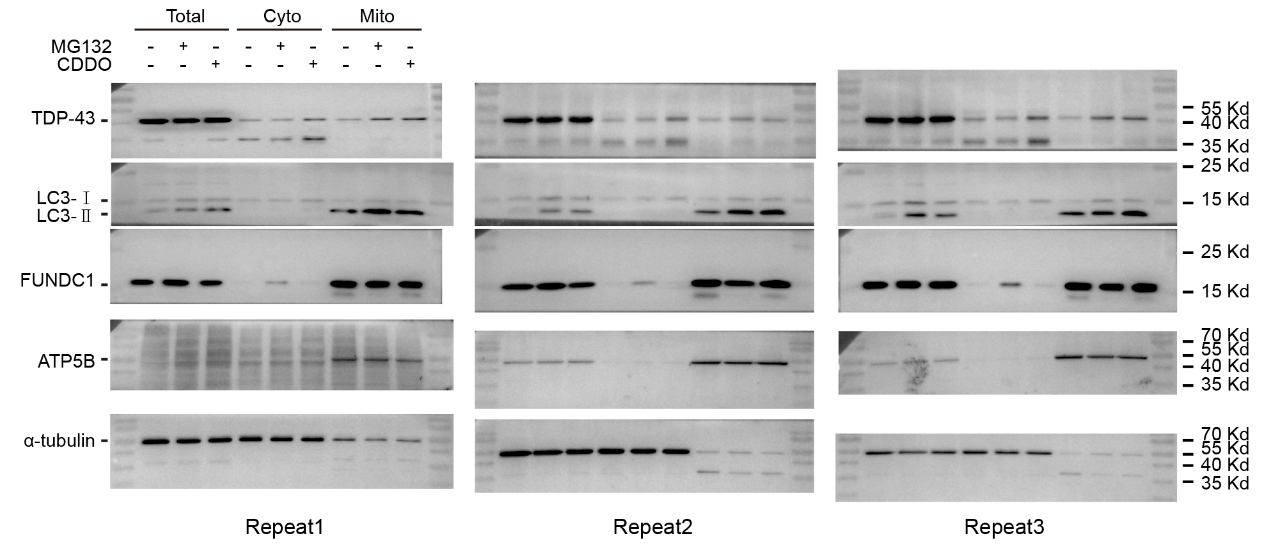


**Source data 13: Inhibition of UPS or LONP1 activates mitophagy.** The results show the source data of Fig S8.
